# CX-5461 causes nucleolar compaction, alteration of peri- and intranucleolar chromatin arrangement, an increase in both heterochromatin and DNA damage response

**DOI:** 10.1038/s41598-022-17923-4

**Published:** 2022-08-17

**Authors:** Luc Snyers, Sylvia Laffer, Renate Löhnert, Klara Weipoltshammer, Christian Schöfer

**Affiliations:** Department for Cell and Developmental Biology, Center for Anatomy and Cell Biology, Medical University of Vienna, Schwarzspanierstr. 17, 1090 Vienna, Austria

**Keywords:** Cell biology, Imaging

## Abstract

In this study, we characterize the changes in nucleolar morphology and its dynamics induced by the recently introduced compound CX-5461, an inhibitor of ribosome synthesis. Time-lapse imaging, immunofluorescence and ultrastructural analysis revealed that exposure of cells to CX-5461 has a profound impact on their nucleolar morphology and function: nucleoli acquired a compact, spherical shape and display enlarged, ring-like masses of perinucleolar condensed chromatin. Tunnels consisting of chromatin developed as transient structures running through nucleoli. Nucleolar components involved in rRNA transcription, fibrillar centres and dense fibrillar component with their major constituents ribosomal DNA, RNA polymerase I and fibrillarin maintain their topological arrangement but become reduced in number and move towards the nucleolar periphery. Nucleolar changes are paralleled by an increased amount of the DNA damage response indicator γH2AX and DNA unwinding enzyme topoisomerase I in nucleoli and the perinucleolar area suggesting that CX-5461 induces torsional stress and DNA damage in rDNA. This is corroborated by the irreversibility of the observed altered nucleolar phenotypes. We demonstrate that incubation with CX-5461, apart from leading to specific morphological alterations, increases senescence and decreases cell replication. We discuss that these alterations differ from those observed with other drugs interfering with nucleolar functions.

## Introduction

Modification of rRNA synthesis rate can occur in response to internal and exogenous stimuli. Many pathways converge on the nucleolus to regulate rRNA synthesis rate and thereby the amount of ribosomes available for protein synthesis. Deregulation, frequently hyper-activation of rRNA synthesis, is a hallmark of cancerogenesis. Developmental differentiation processes often display consecutive stages of up- and downregulation of rRNA synthesis while persistent reduction of rRNA synthesis levels occurs when cells undergo cellular senescence during aging processes^1^.

Ribosomal RNA genes are present as multiple tandem arrays separated by intergenic spacer sequences. In the human genome, rDNA localizes to ten nucleolar organizer regions (NORs). Only a subset of these genes are transcriptionally active. Ribosomal RNA transcription depends on RNA polymerase I (pol I) that requires a set of basal transcription co-factors including the SL1 (selective factor 1) complex to assemble the pre-initiation complex (PIC) at the promoters of rRNA genes^2,3^. The typical mammalian nucleolus consists of three ultrastructural components: strands of dense fibrillar component (DFC) surround the fibrillar centres (FCs) and both fibrillar components are embedded in the granular component (GC). rRNA transcription is thought to occur at the interface between FC and DFC^4,5^ whereas silenced rDNA is located adjacent to the nucleolar surface^6^. Further rRNA processing and ribosome subunit assembly takes place in the GC.

Inhibition of rRNA transcription is an important tool to study nucleolar functions. The toolbox of rRNA transcription inhibitors encompass drugs acting at different levels of transcriptional regulation^7,8^. In experimental studies, a limited set of these drugs is used as inhibitors of rRNA production. The well-established antibiotic actinomycin D (AMD or ActD) and the purine nucleoside analogue DRB (5,6-dichlorobenzimidazole 1-β-D-ribofuranoside) display a predominantly nucleolar effect causing spatial segregation of the three nucleolar components and formation of typical nucleolar caps at the periphery of nucleoli (AMD)^9–11^ or nucleolar disruption and formation of necklace-like nucleoli (DRB)^12^.

Recently, several small molecule inhibitors primarily designed for anti-cancer treatment were reported to reduce rRNA transcription rates^13,14^. Amongst these, the small molecule inhibitor CX-5461^15,16^ has been used both as tool for basic research on nucleolar functions and in clinical trials as anticancer drug. The mode of action of CX-5461 is currently not fully understood. CX-5461 was initially reported to act specifically on pol I by binding to SL1 thereby disrupting PIC formation and preventing binding of pol I to the rDNA gene promoter^15^. Very recently, the specificity of CX-5461 for pol I was challenged by two studies identifying DNA topoisomerase II alpha (topo IIα) as the main effector of CX-5461^17,18^ suggesting a genome-wide effect of CX-5461. In addition, CX-5461 has been associated with stabilization of G-quadruplex DNA (G4) structures^19,20^. G4 structures occur at several genome loci including rDNA gene promoters and intergenic spacer rDNA sequences^21^ and can lead to DNA double strand breaks (DSBs). It was demonstrated that CX-5461 induces DSBs^20^ and acts as mutagen in C. elegans^22^. In consequence of the introduced DSBs, the essential DNA repair pathway ATM/ATR is activated by CX-5461^23,24^. Noteworthy, ATM kinase acts synergistically by initiation of DNA-damage repair response and the direct inhibition of pol I transcription^25^. Further important factors involved in pol I mediated transcription are topoisomerase I and II alpha enzymes. Topoisomerase I (topo I) binding has been identified at various sites in the rDNA, associates with pol I facilitating PIC formation and is thought to alter the topology of the promoter in order to enhance rRNA synthesis and furthermore inhibition of topo I interferes with rRNA transcription^3,26–28^. Topo IIα promotes rRNA transcription by inducing topological changes at gene promoters.

Exposure of cells to established inhibitors AMD and DRB leads to well-documented, substantial changes in nucleolar morphology such as nucleolar segregation (AMD) and disruption (DRB). A detailed description of CX-5461- induced changes is so far lacking. Here, we provide a characterization of rearrangement of nucleolar components induced by CX-5461 in cultures of cancer cells and we use time-lapse microscopy to track the dynamics of nucleolar changes during the exposure of cancer cells to CX-5461. Furthermore we discuss the impact of these changes on nucleolar functionality.

## Results

### CX-5461 incubation reduces rRNA synthesis and induces growth arrest and senescence in HeLa cells

**Figure 1:**
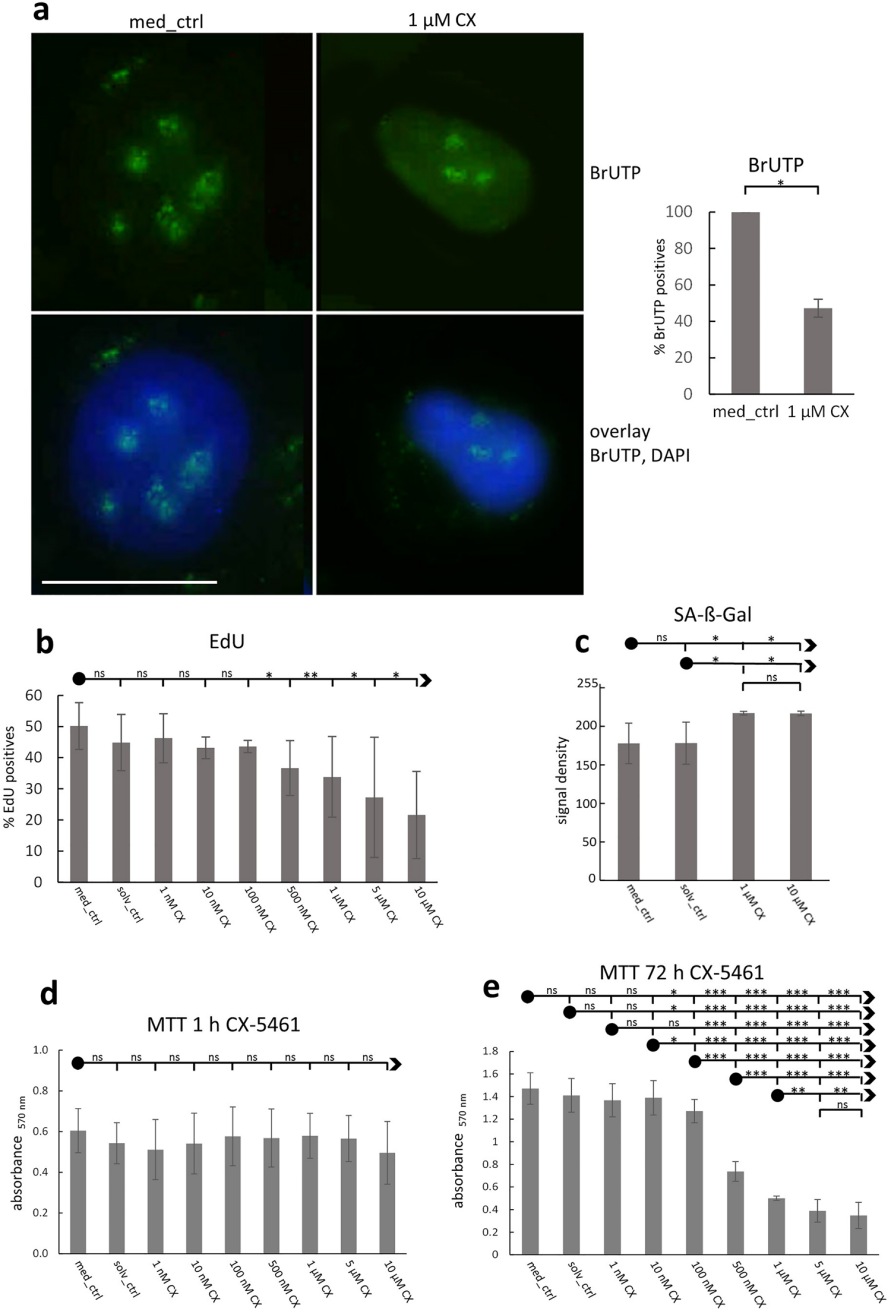
Effect of CX-5461 exposure on HeLa cell cultures. (**a**) Reduced amount of newly synthesized rRNA (green) at 1 µM CX-5461 exposure for 1 h in comparison to control cells visualized by BrUTP-incorporation into nascent transcripts (representative images; widefield imaging); chart shows percentage of BrUTP-positives among a total of 220 cells in 1 experiment; (**b**) 1 h exposure shows significant reduction of cells undergoing DNA replication beginning from 500 nM CX-5461 onwards measured by EdU incorporation (mean 7600 cells per category, 3 independent experiments); statistics: bullet is always used as anchor point for comparison (read: first “ns” compares med_ctrl with solv_ctrl, second “ns” compares med_ctrl with 1 nM CX, etc.); (**c**) senescence is significantly induced at exposure to 1 µM CX-5461 for 1 h as measured by SA-ß-Gal staining (mean of 7200 DAPI-stained nuclei per category (8-bit grey values); 2 independent experiments); (**d**) viability shows no significant differences after 1 h CX-5461 incubation (1 nM–10 µM) measured with an MTT assay whereas incubation for 72 h (**e**) leads to significantly reduced viability, particularly from 500 nM CX-5461 onwards (**d**,**e**: 3 independent experiments (triplicates)). Graph bars display means, error bars represent standard deviations; Student´s *t*-test was applied. Bar = 10 µm, *ns* not significant, *P < 0.05, **P < 0.01, ***P < 0.001.

We used HeLa cells as representative cancer cells commonly utilized in basic research. In order to test the influence of CX-5461 on nucleoli cells were incubated for one hour with varying concentrations of CX-5461, ranging from 1 nM, 10 nM, 100 nM, 500 nM, 1 µM, 5 µM to 10 µM. We considered conditions suitable if the following criteria were fulfilled: onset of altered nucleolar morphology, reduced rRNA synthesis rate, reduced DNA replication, and sustained viability for further analysis. We determined exposure of HeLa cells to 1 µM CX-5461 for 1 h as optimal condition in our experiments. Using this condition, we detected reduced nucleolar transcription by investigating incorporation of BrUTP into nascent rRNA transcripts (Fig. 1a). Furthermore, we found concentration-dependent reduction of DNA replication using EdU incorporation assay (Fig. 1b) and induction of cellular senescence indicated by senescence-associated-ß-galactose (SA-ß-gal) staining (Fig. 1c). Incubation with CX-5461 for one hour did not substantially reduce cellular viability measured with an MTT assay (Fig. 1d) whereas incubating for 72 h led to significantly (P < 0.001) reduced viability starting from a CX-5461 concentration of 500 nM onwards (Fig. 1e). The concentration and incubation time of CX-5461 used in this study, 1 µM for 1 h, is comparable with those of other studies and is lower than the plasma concentration used in clinical studies^29^.

### CX-5461 treatment causes dynamic alterations of nucleolar morphology

**Figure 2:**
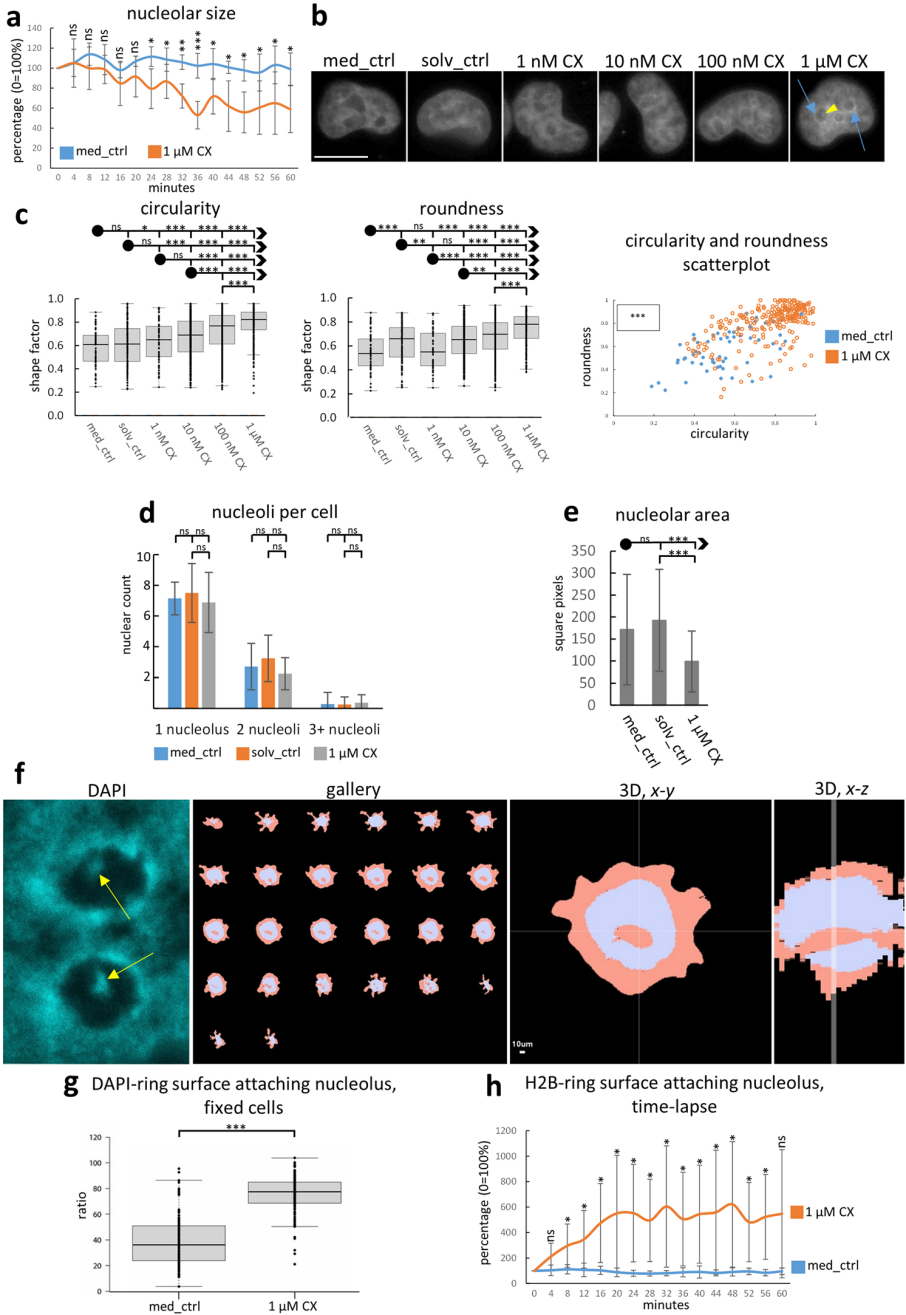
Changes of nucleolar morphology after CX-5461 exposure. (**a**) Time-lapse imaging for 1 h reveals significant nucleolar size reduction of HeLa cells expressing H2B-mCherry beginning after approx. 20 min after exposure to CX-5461 (timepoint 0 = 100% of nucleolar area (each 5 cells out of 1 experiment)). (**b**) DAPI staining shows changes of nucleolar morphology in fixed HeLa cells (representative images of several independent experiments; widefield imaging); note the distinct DAPI-positive ring after 1 µM CX-5461 treatment (blue arrows); note nucleolar dot (yellow arrowhead); (**c**) morphology factors roundness and circularity approach value 0.8 with increasing concentrations of CX-5461 (fixed HeLa cells); 1.0 is indicative of a perfect sphere; scatterplot for visual overview of the shape distributions (a mean of 282 nucleoli per category were evaluated from 3 independent experiments); (**d**) CX-5461 incubation does not show a significant change in the number of nucleoli per cell (counting of the number of cells containing 1, 2 or 3 and more nucleoli per cell; Hep3B cells; a mean of 64 cells with nucleoli per category were evaluated for 3 repeated measurements from 1 experiment); (**e**) the nucleolar size significantly decreases upon CX-5461 treatment (HeLa cells; a mean of 48 nucleoli per category were evaluated from 1 experiment); (**f**) frequently, DAPI-positive dots appear inside nucleoli after 1 µM CX-5461 exposure for one hour in fixed cells (yellow arrows; single confocal section); 3-D reconstruction of nucleolar interior (grey) and perinucleolar chromatin (skin-colour) reveals tunnel-like nature of chromatin strands running through nucleoli (representative HeLa cells with tunnels). (**g**) significant increase of nucleolar surface (perimeter) coverage by the bright DAPI positive perinucleolar heterochromatin upon CX-5461-treatment (fixed HeLa cells; a mean of 45 nucleoli per category evaluated from 2 independent experiments); (**h**) likewise, time-lapse imaging for 1 h revealed significant increase in perinucleolar heterochromatin upon CX-5461 treatment evaluated by the bright perinucleolar signal in H2B-YFP expressing cells; significant differences can be observed after approximately 10 min (each 9 cells out of 1 experiment); bar and line graph display means, error bars represent standard deviations; box plot charts show first and third quartile, sample median and whiskers (1.5× interquartile range); Student´s *t*-test was applied. Bars = 1 µm (**f**), 10 µm (**a**); *ns* not significant, *P < 0.05, **P < 0.01, ***P < 0.001.

We evaluated nucleolar changes using time-lapse microscopy to study the dynamics during incubation of cells with CX-5461 and complemented these data with immunofluorescence analysis on fixed cells to obtain larger sample sizes. Time-lapse imaging of untreated HeLa cells stably expressing either histone H2B-YFP or histone H2B-mCherry showed typical variance of nucleolar shapes present in HeLa cells ranging from large decompacted to small roundish shapes. In controls, the shapes of these nucleoli did not change substantially when cells were recorded for 1 h. In contrast, treatment with 1 µM CX-5461 displayed significant nucleolar changes towards smaller, compacted and roundish nucleoli (P < 0.05). This alteration of nucleolar shapes became apparent after 15 to 20 min exposure to CX-5461 and these significant differences in comparison to control cells persist until 1 h of incubation (Fig. 2a; Supplementary Movie 1). We then analysed nucleolar morphology after 1 h of incubation with CX-5461 in fixed HeLa and Hep3B cells. After treatment with 1 µM CX-5461 for 1 h, nucleoli mirrored the results of time-lapse imaging and nucleoli predominately acquired a compact and spherical shape in DAPI-stained images (Fig. 2b,c) and in images depicting rRNA (Supplementary Fig. 1c) while the number of nucleoli per cell did not change significantly showing that there is no fragmentation of nucleoli (Fig. 2d). However, the nucleolar area per cell was significantly reduced (Fig. 2e; P < 0.001) reflecting the reduced rRNA synthesis rate.

An interesting observation was the appearance of one or more DAPI-positive dots frequently appearing inside nucleoli of fixed cells after CX-5461 incubation (Fig. 2b). 3-D reconstructions based on confocal layers showed that these dots represent cross-sections of tunnels continuous with perinucleolar chromatin (Fig. 2f). Time-lapse imaging revealed that these tunnels are dynamic in nature showing appearance and disappearance during the observation period of 1 h (Supplementary Movie 2).

### CX-5461 induces enlargement of perinucleolar heterochromatin and causes enrichment of heterochromatin markers in nucleoli and perinucleolar region

We noticed that the perinucleolar condensed chromatin was markedly enlarged after CX-5461 exposure. This was detectable as prominent DAPI staining in fixed cell samples and as bright perinucleolar rim in cells stably expressing histone H2B-YFP that appear as distinct and compact ring-like structure around nucleoli resulting in an increased relation of perinucleolar condensed chromatin area to nucleolar area (Fig. 2g,h; Supplementary Movie 1). Subsequently, we addressed the question of the chromatin nature of the perinucleolar condensed chromatin and stained cells with antibodies against the repressive chromatin marks H3K27me3 and H3K9me2 and against DNA methylation (m5C). Additionally, H3K9me2/3-positive chromatin is enriched in AT-rich DNA sequences that are preferentially stained with DAPI. All three markers showed a significant increase of label density over nucleoli and the histone modification markers also over perinucleolar chromatin upon treatment with CX-5461 (P < 0.001; Supplementary Figs. 2a-c; 3a). Colocalization with DAPI revealed extended overlap of both signals indicative of heterochromatin. Interestingly, some stretches showed bright DAPI stain but low intensity of H3K27me3 demonstrating differences within the DAPI-positive ring with respect to chromatin status. Correlation with FISH for rDNA shows that only a minor part of the DAPI-positive ring consists of rDNA sequences (Supplementary Fig. 2a; 3b).

**Figure 3:**
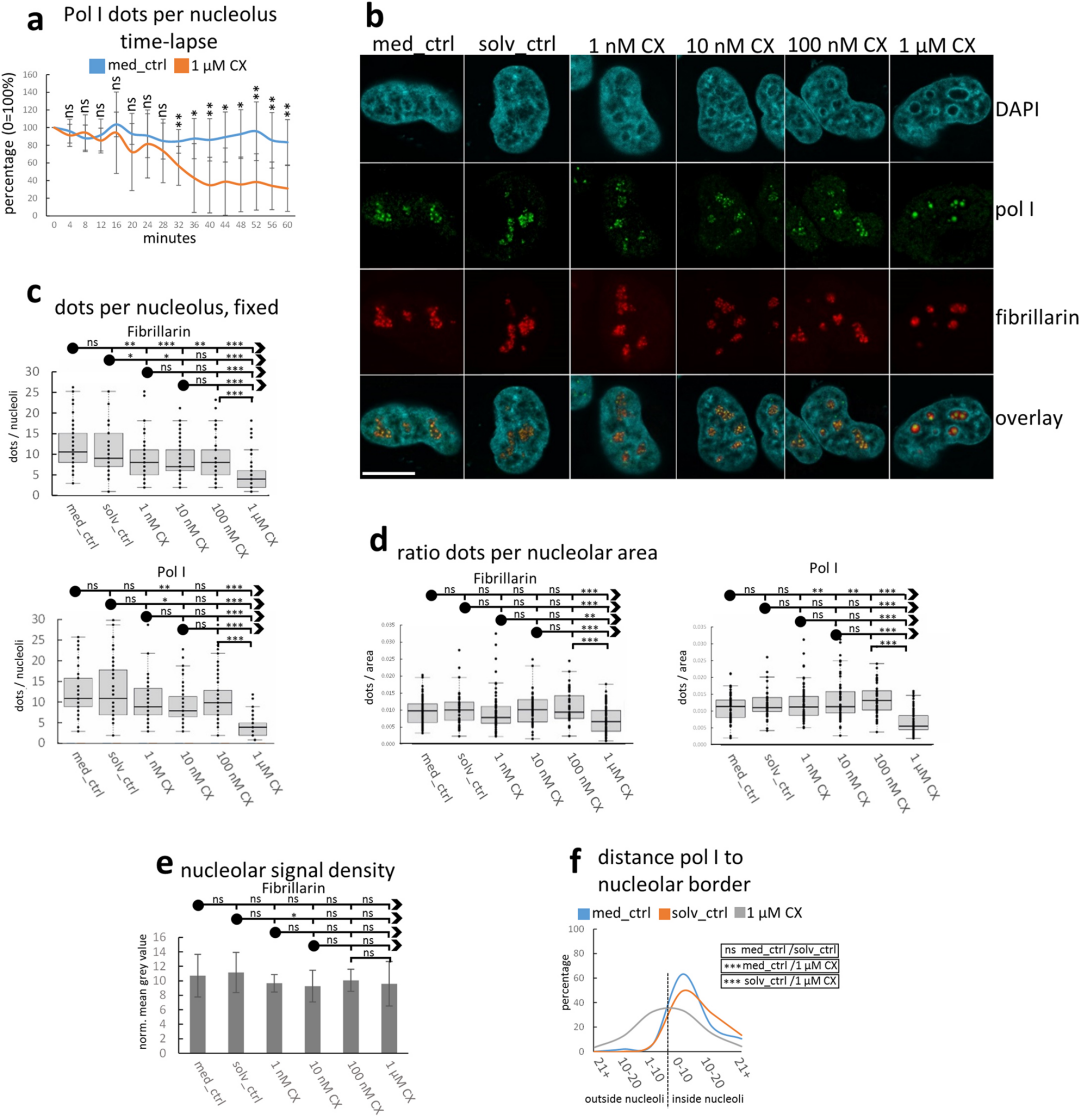
Morphological changes of localization of prominent markers for nucleolar functions upon CX-5461 treatment; HeLa cells. (**a**) Time-lapse imaging over 1 h demonstrates drastic reduction of the number of pol I dots per nucleolus in HeLa cells expressing H2B-mCherry and pol I-GFP upon CX-5461 incubation (each 6 cells out of 1 experiment); (**b**) in fixed cells, both fibrillarin and pol I show abundant foci per nucleolus in control cells and few bright spots in 1 µM CX-5461- treated cells (representative cells; single confocal sections); (**c**) graphs show the decline in foci numbers per nucleolus in fixed cells with increasing concentration of CX-5461; (a mean of 16 nucleoli per category was evaluated in 3 independent experiments); (**d**) graphs indicate the relation of dot number to nucleolar area in fixed cells declining at 1 µM CX-5461; (a mean of 16 nucleoli per category was evaluated in 3 independent experiments) (**e**) the mean fibrillarin intensity per nucleolus does not change significantly at 1 µM CX-5461 in fixed cells (a mean of 16 nucleoli per category was evaluated in 1 experiment); (**f**) graph demonstrating shift of pol I-foci towards nucleolar periphery after incubation with 1 µM CX-5461 as evaluated in fixed cells; dashed line indicates nucleolar border; pixel categories given on x-axis (e.g. 0 to 10 pixels inside the nucleolar border) and percentage of total per sample at the ordinate; statistical evaluation represents summed dots located inside and outside nucleolar border (a mean of 169 pol I signal dots per category was evaluated in 3 independent experiments). Bar and line graphs display means, error bars represent standard deviations; box plot charts show first and third quartile, sample median and whiskers (1.5× interquartile range); Student’s *t*-test was applied except in (**f**) (Chi-square test). Bar = 10 µm; *ns* not significant, *P < 0.05, **P < 0.01, ***P < 0.001.

We complemented our morphological evaluation by analysing the distribution of prominent nucleolar marker molecules for rRNA transcription and structural components. Fibrillarin is involved in rRNA transcription as well as in co-transcriptional processing and serves as a marker for DFC^30,31^. Pol I occurs in high concentrations in FCs^32^. Time–lapse imaging of untreated HeLa cell samples co-expressing histone H2B-mCherry with pol I-GFP as well as of samples co-expressing histone H2B-YFP with fibrillarin-mCherry showed that the numbers of foci of pol I and fibrillarin within nucleoli remain constant throughout 1 h of imaging. In contrast, exposure to CX-5461 led to a significant reduction of nucleolar foci numbers (P < 0.05; Fig. 3a; Supplementary Movies 3 and 4). At the end of the observation period of 1 h we found that some nucleoli had entirely lost their pol I as well as fibrillarin signal indicating transcriptional shut-down in these nucleoli. Co-staining of fibrillarin and pol I in fixed control cells showed that numerous pol I-positive dots are distributed throughout nucleoli. These dots are surrounded by fibrillarin, together forming fibrillar complexes. Strands of fibrillarin also emanate away from the fibrillar complexes (Fig. 3b). In CX-5461- treated cells both markers become restricted to few bright foci within nucleoli and fibrillarin extensions were reduced (Fig. 3b,c). The reduction of both markers was also significant when foci number was related to nucleolar area after treatment with 1 µM CX-5461 (P < 0.001; Fig. 3d). In case of fibrillarin, the appearance of few bright foci after 1 µM CX-5461 treatment was not accompanied by significant changes in mean signal intensities per nucleolus (Fig. 3e). In addition, measuring pol I centre-of-gravity distance towards the nucleolar border in fixed cells revealed a significant relocation towards the nucleolar periphery upon CX-5461 exposure (P < 0.001; Fig. 3f). Prolongation of incubation time in CX-5461 to 72 h aggravated morphological changes (nucleolar shape, pol I-occupancy, perinucleolar heterochromatin) stronger at high than at lower concentrations of CX-5461 (Supplementary Fig. 1a) corroborating the MTT-data (Fig. 1d,e). Nucleoli treated for 72 h with 1 µM CX-5461 showed pronounced localization of fibrillar complexes at the nucleolar periphery accompanied by increased disentanglement of fibrillarin and pol I signal. The mass of fibrillarin is present as strands located inside nucleoli without attaching pol I foci. Few fibrillarin strands are embedded inside the pol I accumulations contrasting the arrangement seen in normal nucleoli and in most 1 h-treated nucleoli (Supplementary Fig. 1b). At the site of the pol I cap the appearance of the perinucleolar chromatin seems to be altered towards a lower state of condensation shown by DAPI staining.

We then detected ribosomal DNA by FISH. The rDNA signal located inside nucleoli of untreated cells, considered to represent largely actively transcribed rRNA genes, assumed a more peripheral localization after CX-5461 treatment mirroring pol I relocation characteristics. Interestingly, FISH for rDNA revealed differences in the composition of the DAPI-positive perinucleolar chromatin (see H3K27me3 data above) and of the chromatin within nucleolar tunnels, both of which harbour ribosomal and non-ribosomal DNA (Supplementary Figs. 2a; 3b).

Summing up, we could show that CX-5461 induces a concentration-dependent reduction of rRNA synthesis and diminished nucleolar volume, which is accompanied by nucleolar compaction and relocalization of the nucleolar constituents, fibrillarin, pol I and rDNA towards the nucleolar periphery.

### Nucleolar alterations induced by CX-5461 are cell-cycle dependent

Next, we tested for possible difference in cell-cycle sensitivity of CX-5461. Cultures of HeLa cells were synchronized by double-thymidine block arresting cells at the G1/S boundary (details in Material and Methods). Released cells were allowed to grow for 1 h, 6 h, and 11 h resulting in samples enriched in S-, G2-, and G1-phase cells. Immediately after the post-release growth period cells were exposed to 1 µM CX-5461 treatment for 1 h. Subsequently, cells were fixed and analysed by fibrillarin and DAPI-staining. A significant decrease of nucleolar size and a reduction of fibrillarin-positive dots were consistently observed after incubation with CX-5461 in G1- and S- phase (P < 0.05; Supplementary Fig. 4a–c). Interestingly, a significant difference in relation of fibrillarin dot number to nucleolar size was observed between exposure of cells to CX-5461 in G2 and G1- phase (P < 0.05; Supplementary Fig. 4d). The combined datasets of the cell cycle stages result in a significant decrease of this ratio after CX-5461 exposure comparable to the evaluation of unsynchronized cells (P < 0.01; Supplementary Fig. 4e). Together, these results indicate a cell cycle-dependent susceptibility variation to treatment with CX-5461.

### CX-5461 induced nucleolar changes are irreversible

We then addressed reversibility of the observed nucleolar phenotype by letting cells grow in normal growth medium (“wash-out” experiment) for 3, 6 and 20 h after CX-5461 incubation before fixation. We found that nucleolar morphology and rDNA localization did not revert to the initial phenotype even after 20 h in normal growth medium (Supplementary Fig. 5a–c). Hence, we conclude that the nucleolar phenotype is irreversibly altered after incubation with 1 µM CX-5461 for 1 h. We noted that cell viability was markedly reduced (Supplementary Fig. 5d). The fact that the nucleolar alterations persist is in line with a DNA-based structural impairment of rRNA synthesis induced by CX-5461, such as the formation of G4 structures or R-loops that may hinder initiation and progression of rRNA synthesis.

### CX-5461 induces substantial ultrastructural changes

**Figure 4:**
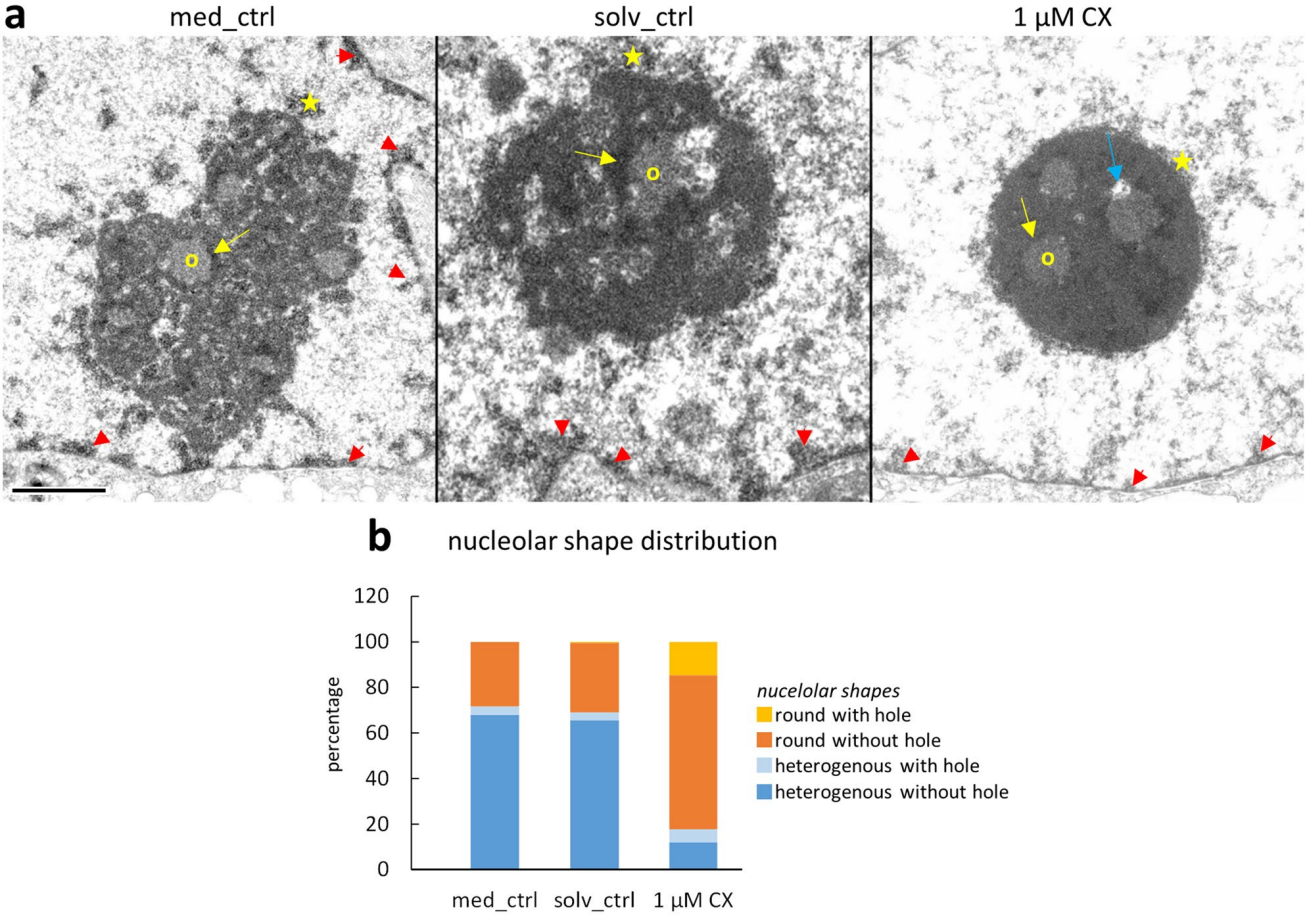
Ultrastructural changes in nucleoli of Hep3B cells treated with CX-5461. (**a**) Representative EM images; left: medium control nucleolus with typical heterogeneous, decompacted shape and several FCs (an example indicated with yellow O) surrounded by DFC (yellow arrow); patches of perinucleolar condensed chromatin (yellow star) are visible. Middle: solvent control nucleolus displays similar architecture as in medium control. Right: typical nucleolus after exposure to 1 µM CX-5461 for 1 h. Note compacted and rounded nucleolus and intranucleolar “holes” visible between FCs and DFCs (blue arrow); the perinucleolar chromatin is present as a continuous layer around the nucleolus (yellow star); the remnant nucleoplasm is less heterochromatic including the nuclear lamina (red arrowheads); (**b**) counting of nucleolar shapes on semi-thin sections (percentages) demonstrates the significant increase (P < 0.001 for both “with” and “with-out” hole) of the round, compacted shape upon CX-5461 treatment (a mean of 345 nucleoli per category was counted in 1 experiment). Bar = 1 µm.

In order to elucidate nucleolar changes in more detail, we performed ultrastructural analysis (Fig. 4). In control cells the well-described heterogeneous, tri-partite organization of nucleolar components, FC, DFC, GC, was evident and the perinucleolar chromatin showed a low level of condensation. Exposure to 1 µM CX-5461 for 1 h led to considerable ultrastructural nucleolar changes towards a round, compact nucleolar phenotype (Fig. 4a,b). Typically, the DFC concentrated around FCs and the extensions into GC were reduced. The ultrastructure of the DFC appeared more electron-dense than in control cells and contained coarse filaments. Occasionally, the FCs were no longer detectable or were found at the nucleolar periphery contacting the perinucleolar chromatin. The DAPI-positive spots inside nucleoli found at light microscopic level corresponded to intra-nucleolar chromatin areas at electron microscopy level frequently located at the interface between FCs and surrounding DFC. As observed in light microscopy, the ultrastructural appearance of these patches resembled that of perinucleolar chromatin. The very prominent DAPI-positive ring in CX-5461-treated cells at light microscopic level presented itself heterogeneous in EM. The perinucleolar chromatin consisted of patches of condensed chromatin interspersed with areas of decondensed chromatin. In contrast, the ultrastructural appearance of the remnant nucleoplasm, including the nuclear periphery was less condensed after CX-5461 incubation than in controls (Fig. 4a). Noteworthy, an increase in chromatin decondensation is a hallmark of senescent cells^33^.

### CX-5461 induces DNA strand break repair in nucleoli resulting in an increase of topoisomerase I occupancy in nucleoli and the perinucleolar region

**Figure 5:**
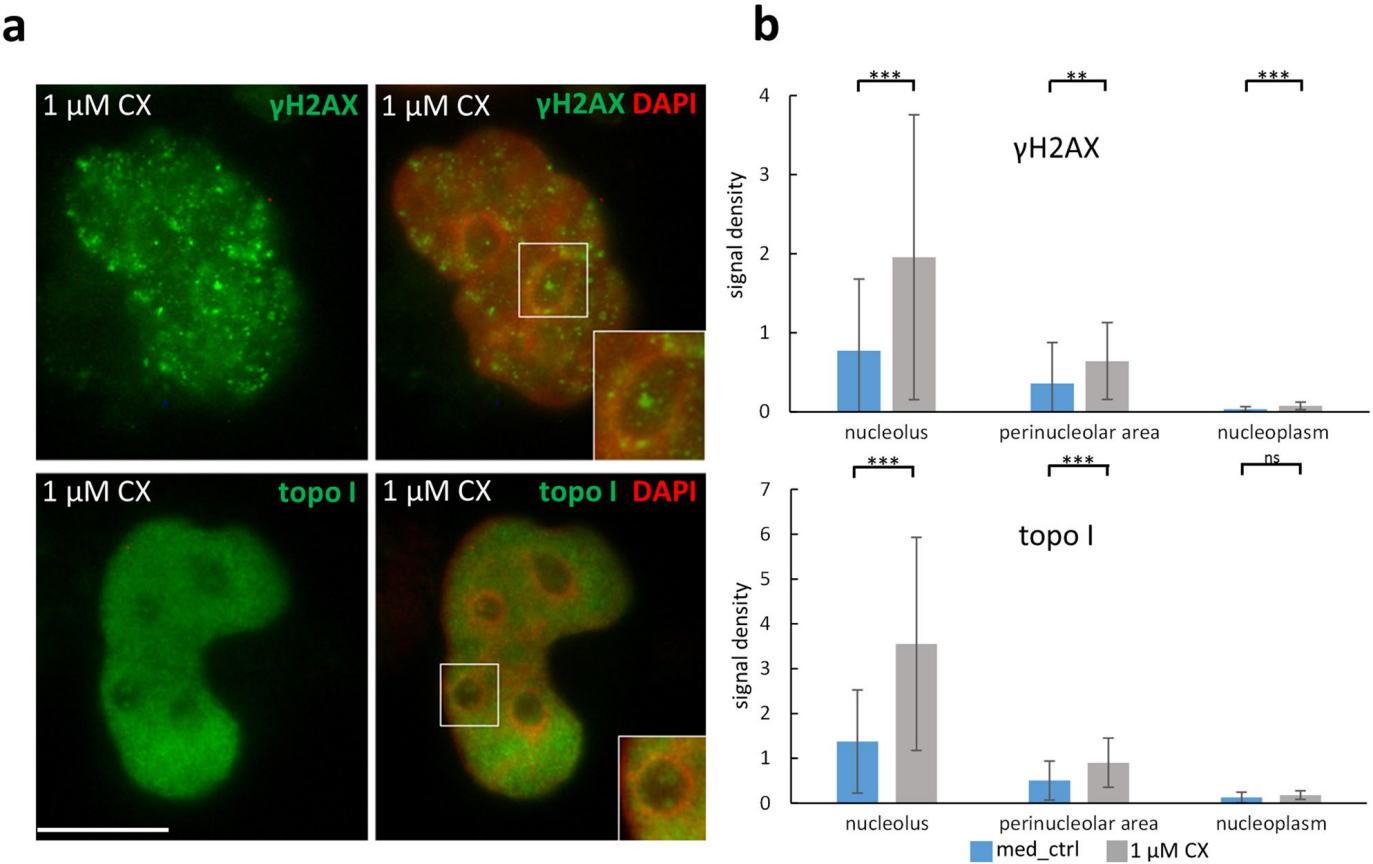
Distribution of γH2AX and topo I after CX-5461 treatment; fixed HeLa cells. (**a**) Representative images of antibody labelling; overlays of IF (green) and DAPI staining (red; false colour); (**b**) γH2AX and topo I show high label denisties over nucleoli and perinucleolar areas after CX-5461 exposure (widefield imaging; 20 randomly chosen nuclei were evaluated in 2 independent experiments each); Graph bars display means, error bars represent standard deviations of mean, background-corrected grey values; Student´s *t*-test was applied. Bars = 10 µm; *ns* not significant, *P < 0.05, **P < 0.01, ***P < 0.001.

Next, we wanted to test if CX-5461 induces DNA damage under the conditions applied. CX-5461 was shown to activate the DNA double strand break (DSB)-dependent ATM/ATR pathway^24,29^ and an increase in DSBs was reported to occur after CX-5461 exposure^20^. Therefore, we performed anti- γH2AX staining, indicative for DSBs (Fig. 5a). Label densities were measured over nucleoli, perinucleloar ring (DAPI-positive ring), and over nucleoplasmic areas. In controls, signal density of γH2AX labelling was present in nucleoli and perinucleolar chromatin and much lower in the nucleoplasm. Treatment with CX-5461 caused a significant increase in signal densities of γH2AX labelling over all three compartments albeit remaining at low level in the nucleolplasm (P < 0.01, P < 0.001; Fig. 5b).

CX-5461 was recently shown to target DNA topoisomerase II alpha^17,18^. Both topo I and topo IIα are present in nucleoli to alleviate torsional stress in rDNA, which led us to hypothesize that CX-5461 might affect topo I distribution in nucleoli. We performed anti-topo I staining (Fig. 5a) and found significant increase of signal density (P < 0.001) in nucleoli and in the perinucleolar region (i.e. the DAPI-positive ring) after CX-5461 exposure, whereas low topo I density was found in the nucleoplasm, which was independent from incubation with CX-5461 (Fig. 5b). Our data suggest that topo I is either recruited to nucleoli as a compensatory reaction to relieve topological stress of rDNA or its DNA binding is stabilized by CX-5461.

In summary, incubation with CX-5461 led to an increased concentration of both γH2AX and topo I signal over nucleoli and perinucleolar chromatin.

## Discussion

Inhibitory drugs that modulate the rRNA synthesis rate are valuable tools to understand basic cell functions in normal and malignant cells. A panel of drugs that reduce rRNA synthesis is available for cancer treatment, most of which used in clinical research^7,8^. The well-established AMD (actinomycin D) acts as a general DNA intercalating agent with a preference for GC-rich sequences such as rDNA. A predominantly nucleolar effect is reached due to the high level of pol I transcription rate. Exposure to higher doses of AMD leads to segregation of the three nucleolar components and formation of typical nucleolar caps at the periphery of nucleoli, which contain rDNA and components of the pol I complex as well as DNA repair factors^9–11^. The purine nucleoside analogue DRB (5,6-dichlorobenzimidazole 1-β-d-ribofuranoside) is a protein kinase inhibitor that affects early steps of ribosomal RNA processing, which occur co-transcriptionally^8,34^. DRB induces nucleolar disruption and formation of necklace-like nucleoli^12^ where each spot is thought to represent one transcribed rRNA gene^35,36^.

None of these drugs show exclusive specificity for factors involved in pol I-driven transcription but affect nucleoli with varying intensity. When initially published, CX-5461 was reported to prevent the pol I basic co-factor SL1 from binding the PIC^16^ and thereby directly affecting rDNA transcription. However, recent reports showed that CX-5461 targets DNA topo IIα function suggestive of a genome-wide primary effect^17,18^. Concerning morphology, our data corroborate the reported dominant effect of CX-5461 on the nucleolus. Further, they show an effect of CX-5461 exposure on the perinucleolar chromatin. At the light microscopic level this is evident by the extent, localization and intensity of DAPI-positive material around the nucleolus. Treatment with CX-5461 leads to an increase of perinucleolar condensed chromatin resulting in the formation of a distinct DAPI-positive ring around nucleoli. The perinucleolar compartment is known to be transcriptionally repressive in nature and is enriched in silenced chromatin (NAD, nucleolus-associated domain). Our data on the increase in signal intensities in the perinucleolar chromatin of the two repressive histone modifications (H3K9me2 and H3K27me3) corroborate this view. In addition, all three markers show an increase of label over the nucleolus after CX- treatment. We believe that this is at least partly due to the appearance of nucleolar tunnels that, at the ultrastructural level and with the markers tested, resemble perinucleolar chromatin. Time-lapse imaging further showed that the increase in perinucleolar heterochromatin as evidenced by H2B expression is quickly induced by CX-5461 within approximately 10 min. Furthermore, nucleoli become compact and spherical in shape upon CX-5461 treatment and the number of fibrillar complexes is reduced after 1 h exposure to CX-5461 in fixed cells. Time-lapse imaging revealed that these nucleolar changes can be detected after about 20 to 30 min exposure to CX-5461. In CX-5461-treated cells the general architecture of the three nucleolar components is largely maintained after 1 h exposure to CX-5461, i.e. the DFC surrounds the FC and both are embedded in the GC. Quantification of fixed samples showed however that these fibrillar complexes move to the nucleolar periphery. The reduction of the number of fibrillar compartments per nucleolus and the observed relocation of fibrillar components and of rDNA to a more peripheral position within nucleoli, i.e. still within GC, correlate with reduced transcriptional activity. Time-lapse imaging further revealed that the reduction of pol I dots induced by CX-5461 is not due to confluency of fibrillar complexes. Rather, we observed a gradual reduction in signal intensity in many foci that finally led to their disappearance. Some of the surviving dots appeared to display higher signal intensities after 1 h exposure to 1 µM CX-5461 than with lower concentrations or shorter exposure times. The higher occupancy of fibrillarin or pol I at these fibrillar complexes suggests compensatory transcriptional upregulation of remaining functional rDNA genes but further studies are required to test this hypothesis. Extending the CX incubation time (1 µM CX-5461) to 72 h explicitly increases the relocation of pol I to the nucleolar periphery where it forms a cap-like structure reminiscent of AMD-treated cells, whereas fibrillarin retains its strand-like conformation within nucleoli. The contact sites between pol I and fibrillarin, considered to be the sites of ongoing rRNA transcription, are reduced (Supplementary Fig. 1b). Fibrillarin no longer surrounds pol I but some fibrillarin strands become engulfed by pol I, which is opposite to normal nucleoli. It can be speculated that these contact sites represent the location of residual rRNA transcription.

**Figure 6:**
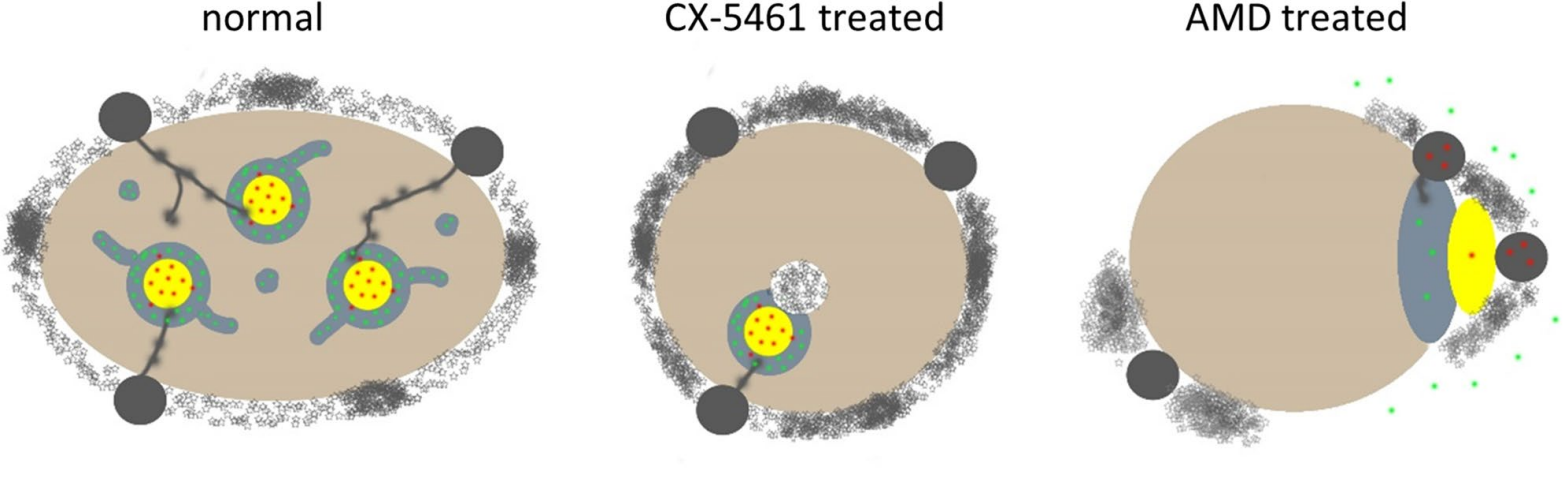
Schematic comparison of morphological changes induced by CX-5461 and AMD treatment. Left: untreated nucleolus (Hela cell): pol I (red dots) containing FCs (yellow) are surrounded by fibrillarin (green dots) containing DFC (grey), which extends into GC (brown); at the nucleolar periphery perinucleolar chromatin consists of stretches of decondensed and condensed chromatin including transcriptionally silent rDNA (NORs; black dots) from which strands of transcriptionally active rDNA emanate into nucleoli. Middle: CX-5461 treatment causes loss of DFC extensions and relocation of fibrillar components to the periphery while maintaining the architecture of FCs and DFCs. Intranucleolar chromatin appears frequently at the interface between FCs and DFCs and the perinucleolar chromatin extends forming a bright DAPI-positive ring around nucleoli, containing mainly condensed chromatin partly including NORs and some stretches of decondensed chromatin. Right: treatment with high doses of AMD resolves FC/DFC architecture causing spatial segregation of the three nucleolar components; rDNA retracts into NORs as does most of pol I while the majority of fibrillarin redistributes into the nucleoplasm; NORs and perinucleolar chromatin appear in patches forming nucleolar caps (scheme compiled with data from^10,59,60^).

Our data show that CX-5461 induces morphological changes different to those after AMD (Fig. 6) or DRB treatment known from the literature. Cells treated with higher doses of AMD display DFC condensation; DFC does not surround FC any more but the two components lie side by side and move to the periphery of the nucleolus. The majority of rDNA is removed from nucleoli and only a minor part remains within DFC^10^. Clearly, CX-5461 does not induce nucleolar fragmentation as is the case with DRB and it does not lead to the complete segregation of nucleolar compartments as is typical for prolonged AMD treatment. Only when cells develop signs of cell death after exposure to high doses of CX-5461 nucleolar segregation eventually occurs. Nucleolar fragmentation has however recently been reported after prolonged exposure to CX-5461 (500 nM for 24 h^37^). It can be speculated that the tendency to move rDNA/fibrillar compartments towards the periphery might be the consequence of retraction of damaged rRNA genes into their respective NORs located outside nucleoli. The fact that nucleolar caps (see below) forming as a consequence of rDNA damage are enriched in DNA repair factors has led to the view that the translocation of rDNA sequences exposes them to nucleoplasm-derived factors required for rDNA silencing and DNA repair^38^.

Nucleolar caps seen after AMD treatment consist of two ultrastructurally distinct entities with an electron dense and an electron lucent appearance^9^. The electron lucent caps contain factors from the fibrillar components such as Pol I, fibrillarin, pre-rRNA and DNA repair factors from the nucleoplasm^7,11,39,40^. Comparing AMD- and CX-5461 exposure, the translocated fibrillar components after 1 h of 1 µM CX-5461 treatment most closely resemble the electron lucent caps although an AMD-like segregation of FCs and DFCs does not occur and the fibrillar components remain embedded within GC albeit a movement towards the nucleolar periphery can be detected. The DAPI-positive ring obtained after CX-5461 exposure would then correspond to the electron dense caps. However, the DAPI positive ring is much more extended than the electron dense caps, appears as a shell surrounding all nucleolar components^39^ and contains NORs and non-ribosomal DNA.

In our study, we find considerable changes in topo I localisation induced by CX-5461. Topo I can be recruited to alleviate torsional stress caused by transcriptional elongation when topo IIα is inhibited as shown for pol II dependent transcription^18^. It is tempting to speculate that similarly the observed increase of topo I density over nucleoli is a compensatory consequence of topo IIα dysfunction. Another contributing factor could be that DSBs induced by G4 stabilization by CX-5461 might extend topo I occupation time at rDNA promoters by stabilizing covalent complexes of topo I and DNA as has been shown for AMD^41^. AMD exposure does not induce DNA damage response in nucleoli^42^, which contrasts with our data of increase of γH2AX triggered by incubation with CX-5461. We found that 1 µM CX-5461 exposure leads to an increase in topo I density in the DAPI-positive ring. For pol II dependent transcription, it has been suggested that inactivation of topo IIα creates DSBs that are refractory to repair^18^. We extend this hypothesis to pol I dependent transcription and propose that damaged rRNA genes move out of nucleoli into NORs where they are permanently silenced. Our data on the irreversibility of the nucleolar changes after CX-5461 incubation (“wash-out”) corroborate this view. In line with these data, it has recently been shown that rDNA sequences carrying DSBs move into nucleolar caps (NORs), which is thought to maintain rDNA integrity by preventing interchromosomal recombination^43^.

The mechanisms driving such a relocation of rDNA are not well understood. The ATM kinase and the MRE11–RAD50–NBS1-complex (MRN complex) facilitate translocation of damaged rDNA sequences to nucleolar caps^44^. Amongst other factors, modification of chromatin and the architecture of rDNA might be important. Actively transcribed rRNA genes are depleted of histones whereas silenced sequences are nucleosomal in nature^45^. It is tempting to speculate that CX-5461-induced silenced rRNA genes become nucleosomal and that post-translational histone modifications of silenced rDNA are involved in the retraction into NORs. Our data on H3K27me3 and H3K9me2 localization across perinucleolar chromatin (Supplementary Figs. 2a,b; 3a) might add to this view. In this respect our findings of the transient formation of intranucleolar tunnels containing ribosomal and non-ribosomal chromatin adds another level of complexity. These tunnels that form between FCs and DFCs might facilitate the access of nucleoplasmic factors (e.g. DNA repair factors) to fibrillar components. In this view the embedment of fibrillar complexes within the body of the nucleolus (i.e. the granular component) might shield transcriptionally active rRNA genes from contact with repressive factors present in the nucleoplasm and in the perinucleolar chromatin that represents a generally repressive nuclear compartment. As pointed out above, migration of fibrillar complexes towards the nucleolar periphery might promote effective silencing of rRNA transcription^38^. The transient appearance of nucleolar tunnels that form between FC and DFC could allow access of repressive factors right to the sites of rRNA transcription, perhaps by lack of nucleolar molecules preventing entry of repressive factors into nucleoli. The structure of these tunnels resembles that of the repressive perinucleolar chromatin, which corroborates this hypothesis. At the same time the tunnels might act as exit sites for silenced rRNA genes as alternative to their translocation to the repressive perinucleolar heterochromatin. Interestingly, the ultrastructural morphology of nucleoli treated with 1 µM CX-5461 is reminiscent of quiescent plant nucleoli^46^. Indeed, we detected increased ultrastructural signs of senescence (Fig. 4) and an increase in senescence using a senescence assay upon CX-incubation (Fig. 1c). This suggests that the observed morphological changes triggered by CX-5461 might at least in part represent a general nucleolar response induced by senescence.

Summing up, we showed that CX-5461 treatment in cultured cells has a profound impact on nucleolar morphology and function of cultured cells. Nucleoli diminish in size and acquire a compact shape surrounded by an increased shell of perinucleolar heterochromatin enriched in repressive chromatin markers. During this process nucleolar tunnels form as transient structures. The transcription-relevant fibrillar complexes reduce in numbers per nucleolus and move to the nucleolar periphery as do rDNA genes. Concomitantly, the extent of perinucleolar condensed chromatin increases along with the occupancy of DNA repair factors. The irreversibility of the nucleolar alterations is in line with an rDNA-based mechanism that causes reduced nucleolar transcription. The observed morphological alterations are accompanied by an increase in senescence and a decrease in cell replication.

## Material and methods

### Cell lines and culture

Hela and Hep3B-p53GFP (named Hep3B in this study^47^) cells were used for assays, immunofluorescence and ultrastructural studies. HeLa cells stably expressing H2B-YFP and H2B-mCherry were used for time-lapse imaging^48^. All cells were cultured in DMEM (Sigma) supplemented with 10% fetal bovine serum (Sigma) and 1% penicillin/streptomycin (Sigma) at 37 °C, 5% CO_2_.

### CX-5461 treatment

CX-5461 (Calbiochem) was dissolved in 50 mM NaH_2_PO_4_ pH  4.5 in 10 mM stocks. Cells were incubated with varying concentrations of CX-5461 in growth medium (1 nM, 10 nM, 100 nM, 500 nM, 1 µM, 5 µM, 10 µM) for 1 h, unless stated otherwise. CX-5461-treated cells were compared to cells grown in growth medium only (“med_ctrl”) and to cells exposed to solvent only (i.e. without CX-5461) in growth medium (“solv_ctrl”).

### Transfection

Cells stably expressing H2B-YFP and H2B-mCherry were transiently transfected with either fibrillarin-mCherry or pol I-GFP using lipofection (Lipofectamine, ThermoFisher) according to the manufacturer´s instructions for HeLa cells. Constructs were obtained from Addgene: the clone of human mCherry-Fibrillarin-C1 was a gift from Susan Janicki (Addgene plasmid # 134539; http://n2t.net/addgene:134539; RRID:Addgene_134539)^49^ and the clone of mouse EGFP-RPA194-C2 was a gift from Tom Misteli (Addgene plasmid # 17660; http://n2t.net/addgene:17660; RRID:Addgene_17660)^50^.

### Antibodies

Primary antibodies: mouse anti-fibrillarin hybridoma supernatant, (^51^, 1: 10), rabbit anti- γH2AX (Millipore anti-phospho H2AX, 1:500), rabbit anti-histone H3K27me3 (Upstate, 1:500), rabbit anti-histone H3K9me2 (Active Motif, 1:1000), mouse anti-5-Methylcytosine antibody (Epigentek, 1:100), human anti-topoisomerase I (^52^, 1:100) and human anti-Pol I (SLR Research Corporation, 1:10). Secondary antibodies: anti-human FITC (Chemicon, 1:100), anti-mouse Alexa488 (Invitrogen, 1:1000), anti-rabbit Alexa488 (Invitrogen, 1:200), anti-mouse Rhodamine F(ab)2 (Chemicon, 1:10), anti-DIG Rhodamine Fab fragments (Roche, 1:200), mouse anti-BrdU (Roche, 1:15).

### BrUTP incorporation assay (visualization of rRNA)

HeLa cells, grown on coverslips overnight in cell growth medium, were washed in PB-buffer, and permeabilized with saponin in PB-buffer for 5 min at 4 °C^53^. Transcription was performed by incubation for 10 min at 33 °C with transcription mix including BrUTP and α-amanitin, or with transcription mix plus solvent reagent or with transcription mix plus CX-5461 (1 µM). Cells were fixed with 2.5% paraformaldehyde and permeabilized with 0.5% Triton X-100 for 2 min. Incorporated BrUTP was detected using the mouse anti-BrdU (Bromodeoxyuridine) antibody that cross-reacts with BrUTP, followed by incubation with the anti-mouse Alexa488 antibody. Cells were counterstained with DAPI and coverslips mounted with Citifluor.

### Viability testing using MTT assay

Metabolic activity was assessed after incubation of HeLa cells with various concentrations of CX-5461 (1 nM, 10 nM, 100 nM, 500 nM, 1 µM, 5 µM, 10 µM) or without the compound (solvent control and medium control) for 1 h and 72 h at 37 °C by addition of 0.5 mg/ml MTT to the medium, followed by an incubation with isopropanol or SDS/HCl. The resulting solute absorbance was measured at 570 nm using an Enspire plate reader (Perkin Elmer) and expressed as mean value of triplicates for each data point including standard deviations.

### DNA replication assay using EdU incorporation

HeLa cells seeded on coverslips were incubated overnight in cell growth medium. Cells were treated with various concentrations of CX-5461 (1 nM, 10 nM, 100 nM, 500 nM, 1 µM, 5 µM, 10 µM) and without the compound (solvent control and medium control) for 1 h. EdU labelling and detection was performed with the Click-iT EdU Imaging Kit, Alexa Fluor 647 dye (Invitrogen) according to the manufacturer´s instructions. Coverslips were counter-stained with DAPI, mounted with Citifluor and evaluated with an automated slide scanning system.

### Senescence-associated ß-galactosidase assay (SA-ß-Gal staining)

HeLa cells grown on coverslips were treated with 1 µM or 10 µM CX-5461 for 1 h and were further incubated for 24 h and 72 h in cell growth medium. Senescence was detected using the Senescence-ß-Galactosidase Cell Staining Kit (Cell Signaling) according to the manufacturer´s protocol. Evaluation was done using an automated slide scanning system.

### Immunofluorescence staining

Cells were seeded onto coverslips or in selected experiments onto uncoated slides and maintained overnight in cell growth medium. Cells were treated with various concentrations of CX-5461 (1 nM, 10 nM, 100 nM, 1 µM) and without the compound (solvent control and medium control) for 1 h unless stated otherwise. Cells were fixed with 4% paraformaldehyde and with methanol. Cells were permeabilized with 0.5% Triton X-100 in PBS for 5 min before blocking with PBST supplemented with 1% BSA and 5% goat serum and incubation with antibodies (primary: overnight, secondary: 30 min). Coverslips were counterstained with DAPI and mounted with Citifluor. For 5-methylcytosine immunofluorescence staining treated cells and controls, fixed with 4% paraformaldehyde, were permeabilized with 1% Triton X-100 in PBS for 1 h at room temperature. Then cells were treated with 4 N HCl 10 min at room temperature, washed with 100 mM Tris–HCl (pH 8.0) for 10 min and with PBS, and were incubated with 0.25 mg/ml or 0.5 mg/ml pepsin for 1 min at 37 °C before blocking as above.

### Probes for FISH (detection of human rDNA and rRNA)

A clone of the EcoRI fragment A encompassing the 5′end of 18S, 5.8S and most of 28S rDNA was used for rRNA detection and the EcoRI fragment D, HindIII sub-fragment DHH from the intergenic spacer region was used for rDNA detection^54^. Clones were labelled with Digoxigenin (DIG) using the DIG-Nick Translation Mix (Roche) according to the manufacturer´s protocol.

### Fluorescence in situ hybridization

Hela cells or Hep3B cells were seeded onto coverslips, incubated overnight in cell growth medium and incubated either with 1 µM CX-5461 or without the compound (solvent control and medium control) for 1 h. FISH was performed as in^55^; briefly, cells were fixed with 4% paraformaldehyde, treated with RNase A (omitted for FISH to detect rRNA), digested with 0.025% pepsin (HeLa) or 0.01% pepsin (Hep3B). Post-fixed, dehydrated and air-dried cells were incubated with the Dig-labelled probes for 7 min at 85 °C and allowed to hybridize overnight at 37 °C. Stringency washes in 2× SSC/50% formamide at 42 °C for 10 min were followed by washes in 2× SSC at 42 °C for 10 min. After blocking (4 × SSCT, 1% bovine serum albumin, 10% sheep serum and 10% goat serum) probes were detected with the anti-DIG rhodamine antibody and coverslips were counterstained with DAPI and mounted with Citifluor.

### “Wash-out” experiment

HeLa cells seeded on coverslips were incubated overnight in cell growth medium. Cells were treated with 1 µM CX-5461 and without the compound (solvent control and medium control) for 1 h. After short washes cells were either immediately fixed (0 h) or further incubated with cell growth medium for 3 h, 6 h or 20 h before fixation with 4% paraformaldehyde.

### Cell cycle analysis (cell synchronization)

HeLa cells were grown on coverslips in cell growth medium with a confluence of 30–50%. Synchronization of cells was performed by double thymidine block to arrest cells at the G1/S boundary^56^. Briefly, cells were incubated in cell growth medium supplemented with 2 mM thymidine for 16 h in a 37 °C CO_2_-incubator; then cells were released for 8 h by incubation in cell growth medium before a second block with 2 mM thymidine for 16 h. Subsequently, cells were released by incubation in cell growth medium for periods of either 1 h, 6 h, or 11 h. That way, our HeLa cell cultures were enriched with cells in S- phase (1 h), G2- phase (6 h), and G1-phase (11 h). Directly after these periods cells were treated with 1 µM CX-5461 or incubated without the compound (solvent control and medium control) for 1 h and immediately fixed with 4% paraformaldehyde.

### Electron microscopy

Hep3B cells seeded in a 6-well plate were incubated overnight in cell growth medium and were treated with various concentrations of CX-5461 (1 nM, 10 nM, 100 nM, 1 µM) and without the compound (solvent control and medium control) for 1 h. Cells were fixed with 2.5% glutaraldehyde in 0.1 M cacodylate buffer for 1.5 h, post-fixed with 1% aqueous OsO_4_, and embedded in Epon 812 according to standard procedures. Ultra-thin sections were cut using an Ultracut S microtome and contrasted with uranyl acetate and lead citrate.

### Imaging

Time-lapse imaging was performed with confocal microscope FV3000 (Olympus) using oil immersion lens 60× (NA 1.25) in a heated incubator hood set to 37 °C. Cells were recorded while growing in closed POCmini cultivation chambers (Pecon). Stained coverslips of fixed cells were recorded with widefield epifluorescence microscope (Eclipse 800, Nikon; 40× (NA 1.0), 60× (NA 1.4) and 100× (NA 1.4)) or with an automated slide scanning system (VS120, Olympus; 40× (NA 1.25)); single confocal sections and stacks were acquired with the confocal microscope (above). Ultrastructural images were obtained with a 1200EXII TEM (Jeol).

### Image software and 3-D reconstruction

Brightness and contrast of images and median filtering (Gaussian blur, 0.8) was applied using Photoshop (Adobe), which was also used to arrange image panels. All image measurements (see Supplementary Methods for applied strategies) and generation of time-lapse movies was done with Fiji^57^. 3-D reconstruction of series of confocal sections were made with the orthogonal view option of Icy^58^.

### Statistics

For most statistics, the two-sided Student’s *t*-test integrated in the spreadsheet program Excel was applied; Excel was also used to generate bar and line graphs and scatter plots. For categorized data (Fig. 3f) a Chi-square test was applied (http://www.socscistatistics.com). Box plots were generated with the online R-tool BoxPlotR (http://shiny.chemgrid.org/boxplotr/). P-values were categorized as ns—not significant, *P < 0.05; **P < 0.01; ***P < 0.001.

## Supplementary Information

Supplementary Information.

Supplementary Video 1.

Supplementary Video 2.

Supplementary Video 3.

Supplementary Video 4.

## Data Availability

The datasets generated during and analysed during the current study are available from the corresponding author upon request.

## References

[1] Tiku V, Antebi A (2018). Nucleolar function in lifespan regulation. Trends Cell Biol..

[2] Heix J (1998). Mitotic silencing of human rRNA synthesis: inactivation of the promoter selectivity factor SL1 by cdc2/cyclin B-mediated phosphorylation. EMBO J..

[3] Hannan RD, Cavanaugh A, Hempel WM, Moss T, Rothblum L (1999). Identification of a mammalian RNA polymerase I holoenzyme containing components of the DNA repair/replication system. Nucleic Acids Res..

[4] Mosgoeller W, Schofer C, Steiner M, Sylvester JE, Hozak P (2001). Arrangement of ribosomal genes in nucleolar domains revealed by detection of "Christmas tree" components. Histochem. Cell Biol..

[5] Yao RW (2019). Nascent pre-rRNA sorting via phase separation drives the assembly of dense fibrillar components in the human nucleolus. Mol. Cell.

[6] Schofer C, Weipoltshammer K (2018). Nucleolus and chromatin. Histochem. Cell Biol..

[7] Bensaude O (2011). Inhibiting eukaryotic transcription: Which compound to choose? How to evaluate its activity?. Transcription.

[8] Burger K (2010). Chemotherapeutic drugs inhibit ribosome biogenesis at various levels. J. Biol. Chem..

[9] Reynolds RC, Montgomery PO, Hughes B (1964). Nucleolar, "caps" produced by actinomycin D. Cancer Res..

[10] Schöfer C, Weipoltshammer K, Almeder M, Müller M, Wachtler F (1996). Redistribution of ribosomal DNA after blocking of transcription induced by actinomycin D. Chromosome Res..

[11] Harding SM, Boiarsky JA, Greenberg RA (2015). ATM dependent silencing links nucleolar chromatin reorganization to DNA damage recognition. Cell Rep..

[12] Granick D (1975). Nucleolar necklaces in chick embryo fibroblast cells. I. Formation of necklaces by dichlororibobenzimidazole and other adenosine analogues that decrease RNA synthesis and degrade preribosomes. J. Cell Biol..

[13] Scull CE (2019). Discovery of novel inhibitors of ribosome biogenesis by innovative high throughput screening strategies. Biochem. J..

[14] Rothblum K, Hu Q, Penrod Y, Rothblum LI (2014). Selective inhibition of rDNA transcription by a small-molecule peptide that targets the interface between RNA polymerase I and Rrn3. Mol. Cancer Res. (MCR).

[15] Drygin D (2011). Targeting RNA polymerase I with an oral small molecule CX-5461 inhibits ribosomal RNA synthesis and solid tumor growth. Cancer Res..

[16] Haddach M (2012). Discovery of CX-5461, the first direct and selective inhibitor of RNA polymerase I, for cancer therapeutics. ACS Med. Chem. Lett..

[17] Bruno PM (2020). The primary mechanism of cytotoxicity of the chemotherapeutic agent CX-5461 is topoisomerase II poisoning. Proc. Natl. Acad. Sci. USA.

[18] Bossaert M (2021). Transcription-associated topoisomerase 2alpha (TOP2A) activity is a major effector of cytotoxicity induced by G-quadruplex ligands. Elife.

[19] Musso L (1862). c-MYC G-quadruplex binding by the RNA polymerase I inhibitor BMH-21 and analogues revealed by a combined NMR and biochemical approach. Biochim. Biophys. Acta Gen. Subj..

[20] Xu H (2017). CX-5461 is a DNA G-quadruplex stabilizer with selective lethality in BRCA1/2 deficient tumours. Nat. Commun..

[21] Rhodes D, Lipps HJ (2015). G-quadruplexes and their regulatory roles in biology. Nucleic Acids Res..

[22] Ye FB (2020). A multimodal genotoxic anti-cancer drug characterized by pharmacogenetic analysis in *Caenorhabditis elegans*. Genetics.

[23] Negi SS, Brown P (2015). rRNA synthesis inhibitor, CX-5461, activates ATM/ATR pathway in acute lymphoblastic leukemia, arrests cells in G2 phase and induces apoptosis. Oncotarget.

[24] Quin J (2016). Inhibition of RNA polymerase I transcription initiation by CX-5461 activates non-canonical ATM/ATR signaling. Oncotarget.

[25] Kruhlak M (2007). The ATM repair pathway inhibits RNA polymerase I transcription in response to chromosome breaks. Nature.

[26] Ray S (2013). Topoisomerase IIalpha promotes activation of RNA polymerase I transcription by facilitating pre-initiation complex formation. Nat. Commun..

[27] Rose KM (1988). Association of DNA topoisomerase I and RNA polymerase I: A possible role for topoisomerase I in ribosomal gene transcription. Chromosoma.

[28] Thomsen B (1987). Sequence specificity of DNA topoisomerase I in the presence and absence of camptothecin. EMBO J..

[29] Sanij E (2020). CX-5461 activates the DNA damage response and demonstrates therapeutic efficacy in high-grade serous ovarian cancer. Nat. Commun..

[30] Tiku V (2018). Nucleolar fibrillarin is an evolutionarily conserved regulator of bacterial pathogen resistance. Nat. Commun..

[31] Iyer-Bierhoff A (2018). SIRT7-dependent deacetylation of fibrillarin controls histone H2A methylation and rRNA synthesis during the cell cycle. Cell Rep..

[32] Scheer U, Rose KM (1984). Localization of RNA polymerase I in interphase cells and mitotic chromosomes by light-and electron microscopic immunocytochemistry. Proc. Natl. Acad. Sci..

[33] Swanson EC, Rapkin LM, Bazett-Jones DP, Lawrence JB (2015). Unfolding the story of chromatin organization in senescent cells. Nucleus.

[34] David-Pfeuty T, Nouvian-Dooghe Y, Sirri V, Roussel P, Hernandez-Verdun D (2001). Common and reversible regulation of wild-type p53 function and of ribosomal biogenesis by protein kinases in human cells. Oncogene.

[35] Haaf T, Ward DC (1996). Inhibition of RNA polymerase II transcription causes chromatin decondensation, loss of nucleolar structure, and dispersion of chromosomal domains. Exp. Cell Res..

[36] Scheer U, Hugle B, Hazan R, Rose KM (1984). Drug-induced dispersal of transcribed rRNA genes and transcriptional products: Immunolocalization and silver staining of different nucleolar components in rat cells treated with 5,6-dichloro-beta-D-ribofuranosylbenzimidazole. J. Cell Biol..

[37] Zhou H (2020). H3K9 demethylation-induced R-loop accumulation is linked to disorganized nucleoli. Front. Genet..

[38] van Sluis M, McStay B (2015). A localized nucleolar DNA damage response facilitates recruitment of the homology-directed repair machinery independent of cell cycle stage. Genes Dev..

[39] Shav-Tal Y (2005). Dynamic sorting of nuclear components into distinct nucleolar caps during transcriptional inhibition. Mol. Biol. Cell.

[40] van Sluis M, McStay B (2017). Nucleolar reorganization in response to rDNA damage. Curr. Opin. Cell Biol..

[41] Trask DK, Muller MT (1988). Stabilization of type I topoisomerase-DNA covalent complexes by actinomycin D. Proc. Natl. Acad. Sci..

[42] Ma H, Pederson T (2013). The nucleolus stress response is coupled to an ATR-Chk1-mediated G2 arrest. Mol. Biol. Cell.

[43] Warmerdam DO, van den Berg J, Medema RH (2016). Breaks in the 45S rDNA lead to recombination-mediated loss of repeats. Cell Rep..

[44] Korsholm LM (2019). Double-strand breaks in ribosomal RNA genes activate a distinct signaling and chromatin response to facilitate nucleolar restructuring and repair. Nucleic Acids Res..

[45] Conconi A, Widmer RM, Koller T, Sogo JM (1989). Two different chromatin structures coexist in ribosomal RNA genes throughout the cell cycle. Cell.

[46] Mineur P, Jennane A, Thiry M, Deltour R, Goessens G (1998). Ultrastructural distribution of DNA within plant meristematic cell nucleoli during activation and the subsequent inactivation by a cold stress. J. Struct. Biol..

[47] Philimonenko AA (2011). Chromosomal dynamics of cell cycle regulator gene p21 during transcriptional activation. J. Struct. Biol..

[48] Snyers L (2014). Distinct chromatin signature of histone H3 variant H3.3 in human cells. Nucleus.

[49] Newhart A (2016). RNase P protein subunit Rpp29 represses histone H3.3 nucleosome deposition. Mol. Biol. Cell.

[50] Dundr M (2002). A kinetic framework for a mammalian RNA polymerase in vivo. Science.

[51] Yang JM, Baserga SJ, Turley SJ, Pollard KM (2001). Fibrillarin and other snoRNP proteins are targets of autoantibodies in xenobiotic-induced autoimmunity. Clin. Immunol..

[52] Mosgoeller W (1998). Ribosomal gene transcription is organized in foci within nucleolar components. Histochem. Cell Biol..

[53] Hozak P, Cook PR, Schöfer C, Mosgöller W, Wachtler F (1994). Site of transcription of ribosomal RNA and intranucleolar structure in HeLa cells. J. Cell Sci..

[54] Gonzalez IL, Sylvester JE (1995). Complete sequence of the 43-kb human ribosomal DNA repeat: Analysis of the intergenic spacer. Genomics.

[55] Wachtler F (1991). Transcribed and nontranscribed parts of the human ribosomal gene repeat show a similar pattern of distribution in nucleoli. Cytogenet. Cell Genet..

[56] Chen G, Deng X (2018). Cell synchronization by double thymidine block. Bio Protoc..

[57] Schindelin J (2012). Fiji: an open-source platform for biological-image analysis. Nat. Methods.

[58] de Chaumont F (2012). Icy: An open bioimage informatics platform for extended reproducible research. Nat. Methods.

[59] Li Y, Hu Y, Che L, Jia J, Chen M (2016). Nucleolar localization of small G protein RhoA is associated with active RNA synthesis in human carcinoma HEp-2 cells. Oncol. Lett..

[60] Jordan P, Mannervik M, Tora L, Carmo-Fonseca M (1996). In vivo evidence that TATA-binding protein/SL1 colocalizes with UBF and RNA polymerase I when rRNA synthesis is either active or inactive. J. Cell Biol..

