# Supplementary material for: CX-5461 causes nucleolar compaction, alteration of peri- and intranucleolar chromatin arrangement, an increase in both heterochromatin and DNA damage response
Source: Sci Rep. 2022 Aug 17;12:13972. doi: 10.1038/s41598-022-17923-4 (PMC9385865; doi:10.1038/s41598-022-17923-4)
Supplement: Supplementary file 1 — Supplementary Information. [file 41598_2022_17923_MOESM1_ESM.pdf]

Supplementary Information

CX-5461 causes nucleolar compaction, alteration of peri- and intranucleolar chromatin arrangement, an increase in both heterochromatin and DNA damage response

Luc Snyers[1,2], Sylvia Laffer[1,2], Renate Löhnert[1], Klara Weipoltshammer[1] and Christian Schöfer[1]*

[1] Department for Cell and Developmental Biology, Center for Anatomy and Cell Biology, Medical University of Vienna, A-1090 Vienna, Austria

[2] Both authors contributed equally

Correspondence (*):

Christian Schöfer
Department for Cell and Developmental Biology
Center for Anatomy and Cell Biology
Medical University of Vienna
Schwarzspanierstr. 17
1090 Vienna Austria
Mail: christian.schoefer@meduniwien.ac.at
Tel.: +43 1 40160 37713
Web: www.meduniwien.ac.at/celldev/

Supplementary Methods

Image-based Quantification. BrUTP positive cells and DAPI-stained nuclei were manually counted on images recorded with the slide scanner (Fig. 1a). For evaluation of cells undergoing DNA replication, images of cells treated with EdU (red channel) and DAPI-stained nuclei (blue channel) were counted using segmentation by grey value thresholding and particle counting function of Fiji (Fig. 1b). Quantification of images with SA-ß-Gal stained cells was done on thresholded transmitted light images and measurement of background-corrected integrated density (pixel sum over a cell) and given as ratio with cell counts based on DAPI-stained nuclei (Fig. 1c). Nucleolar areas were measured by manually outlining borders of nucleoli on DAPI stained images (fixed cells; Fig. 2e) and on consecutive series of cells expressing H2B obtained by live cell imaging (every second image was evaluated; Fig 2a). The shape descriptors "circularity" and "roundness" of the Fiji software were used to quantify changes in nucleolar shape in response to CX-5461 treatment. Nucleoli were manually outlined on DAPI-stained images acquired with a 100x lens (Fig. 2c, Supplementary Figs. 1a; 5c). The extension of bright DAPI-positive perinucleolar chromatin around nucleoli was measured by manual delineation of nucleoli, perinucleolar chromatin and the stretches of contact between nucleolar perimeter and perimeter of the DAPI-positive ring (Fig. 2g). The same strategy was applied for time-lapse imaging where H2B expression was manually evaluated at every second image over the imaging period of 1 hour (Fig. 2h; Supplementary Fig. 1a). Numbers of nucleoli were manually counted on 500 nm thick, toluidine blue-stained semi-thin sections. For each sample all cells of an entire section area were evaluated in three randomly chosen, non-consecutive sections where only those cells were counted that displayed at least one nucleolus (Fig. 2d). Variability of nucleolar shapes was evaluated by manual categorization of nucleoli on semi-thin sections (Fig. 4b). Number of fibrillarin and pol I-dots were manually counted on images of nucleoli taken with a 100x lens (Fig. 3c,d; Supplementary Figs. 1a; 4c) and on image series obtained by life cell imaging on every second image (Fig. 3a). Akin to this approach, fibrillarin and pol I quantifications were made on immunofluorescence images taken with a 100x lens and nucleoli were manually delineated in the DAPI channel using Fiji (Figs. 3 d,e; Supplementary Figs. 1a; 4d,e). In order to measure distances of pol I foci to nucleolar border nucleoli were manually delineated in DAPI channel. A threshold was set for the pol I signal, the closest distances of the signal outline towards the nuclear border was manually measured (Fiji) and the resulting values were categorized as in Fig. 3f. Intensities of topo I, γH2AX, H3K27me3, H3K9me2 and m5C were measured by determination of the background-corrected mean density over determined regions-of-interests (ROIs). ROIs of nucleolar-, perinucleolar- and randomly chosen areas in the nucleoplasm were manually outlined in DAPI channel and used as masks for density measurements in the IF-channels (Fig. 5b; Supplementary Fig. 3a). Cell densities in wash-out experiments were counted on randomly chosen frames of DAPI-stained images (segmented with Fiji) taken with a 60x lens (Supplementary Fig. 5d).

Supplementary Figures

Suppl. Fig. 1

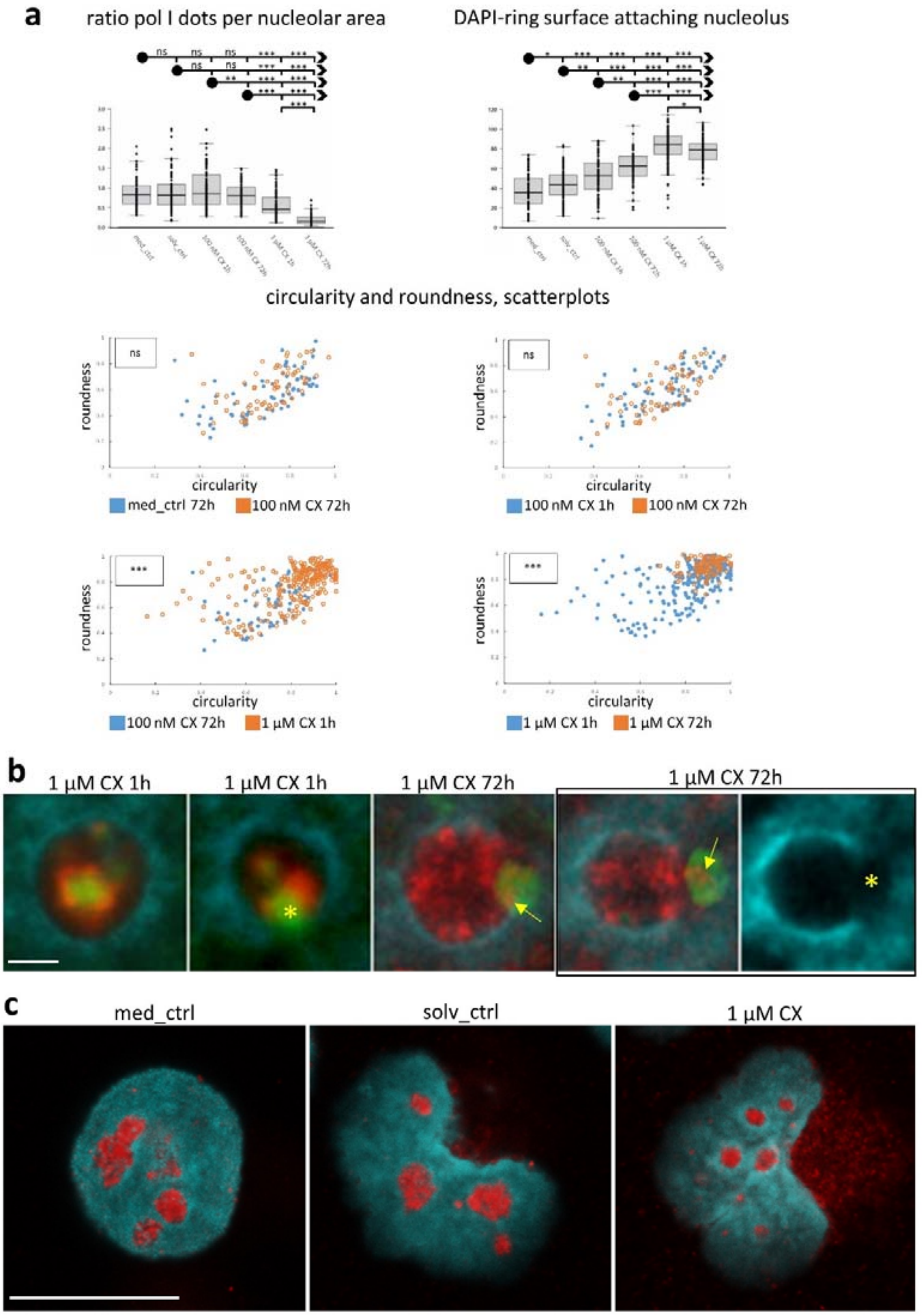

Figure S1. Effects of prolonged incubation time with CX-5461; FISH to detect rRNA; fixed HeLa cells. (**a**) comparison of 1 h and 72 h incubation with 100 nM and 1 µM CX-5461; prolonged incubation time increases morphological changes stronger at higher (1 µM CX-5461) than at lower concentrations (100 nM CX-5461); compare with Figs. 2c,g; 3d; (3 repeated measurements from 2 independent experiments); (**b**) Representative nucleoli (confocal sections) showing pol I (green) and fibrillarin (red) staining after 1 h and 72 h incubation with 1 µM CX-5461 (DAPI blue). Left to right: two typical nucleoli after 1 h, second nucleolus: fibrillar complex close to nucleolar periphery (yellow asterisk); two typical nucleoli after 72 h showing re-arrangement of pol I and fibrillarin with few strands of fibrillarin embedded in pol I-positive material (yellow arrows point to fibrillarin inside pol I) and aggregates of pol I at the nucleolar rim replacing the DAPI-positive chromatin (yellow asterisk; rightmost: DAPI only, same image as to the left). (**c**) rRNA detection (red; FISH) demonstrates decrease in nucleolar size and increase in DAPI-positive perinucleolar heterochromatin (DAPI: blue) after treatment with 1 µM CX-5461 (1 h); confocal sections. Box plot charts show first and third quartile, sample median and whiskers (1.5 x interquartile range); Student´s *t*-test was applied. Bars= 1 µm (b), 10 µm (c); ns= not significant, * $p<0.05$, ** $p<0.01$, *** $p<0.001$.

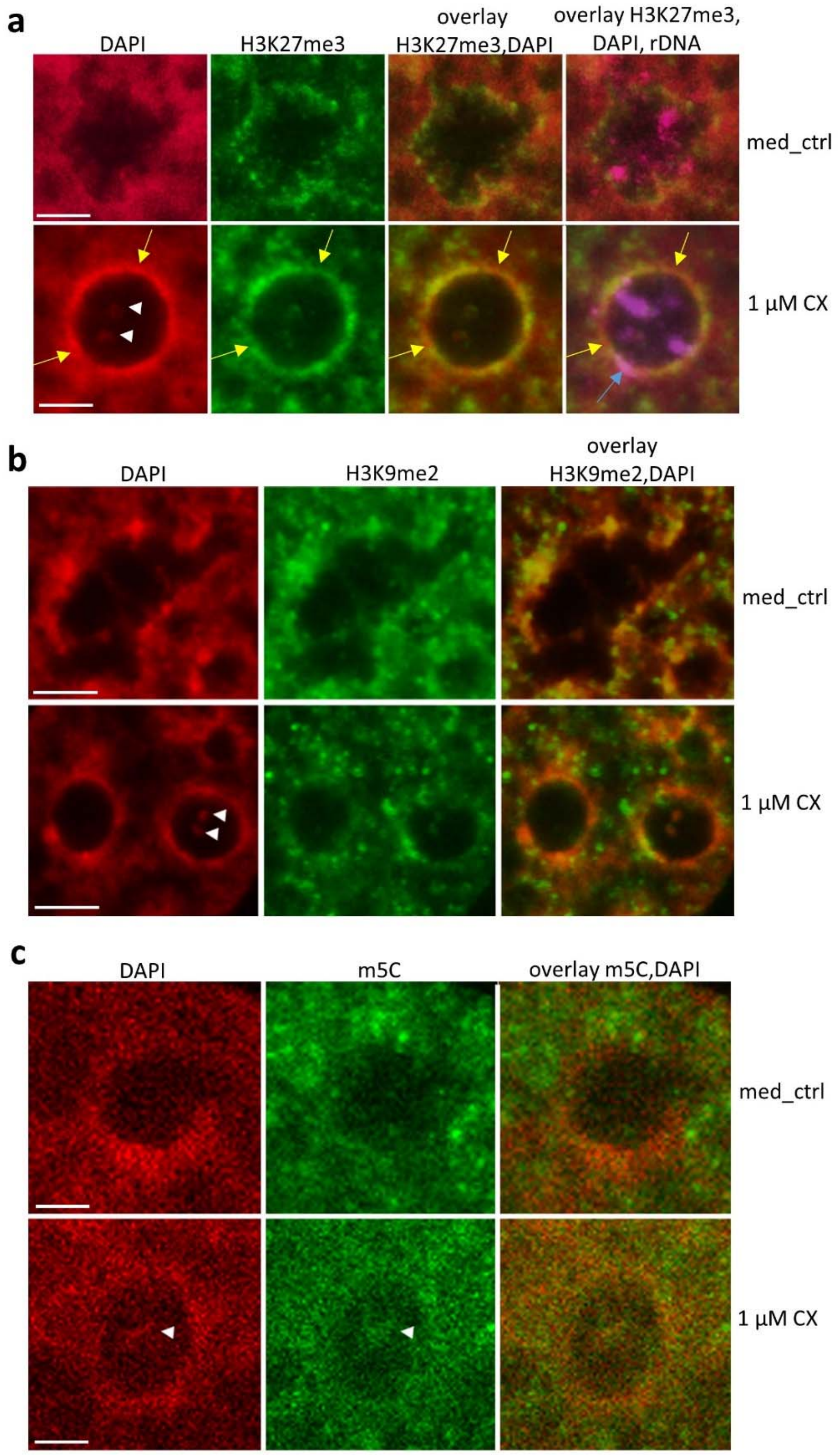

Figure S2. Immunofluorescence to show markers of DNA methylation and heterochromatin in fixed HeLa cells; representative images (confocal sections). (**a**) immunostaining for H3K27me3 (green) shows largely overlapping staining with DAPI (red; false colour) although some stretches appear reduced in H3K27me3 labelling compared to DAPI staining (yellow arrows); overlay with rDNA (FISH, pink) shows that more rDNA signal can be detected in the perinucleolar chromatin of cells incubated with 1µM of CX-5461 than in controls (blue arrow); the perinucleolar chromatin consists of rDNA and non-rDNA sequences; white arrowheads: nucleolar tunnels in CX-5461 treated cells; in this case associated with rDNA. (**b**, **c**) heterochromatin marker H3K9me2 (green) and DNA methylation (m5C; green) immunostaining also demonstrate an increase in nucleolar (both markers)and perinucleolar (H3K9me2) labelling in CX treated cells. All markers are present in the intranucleolar tunnels (white arrowheads). Single confocal sections; for quantification see Figure S3. Bar= 1 µm

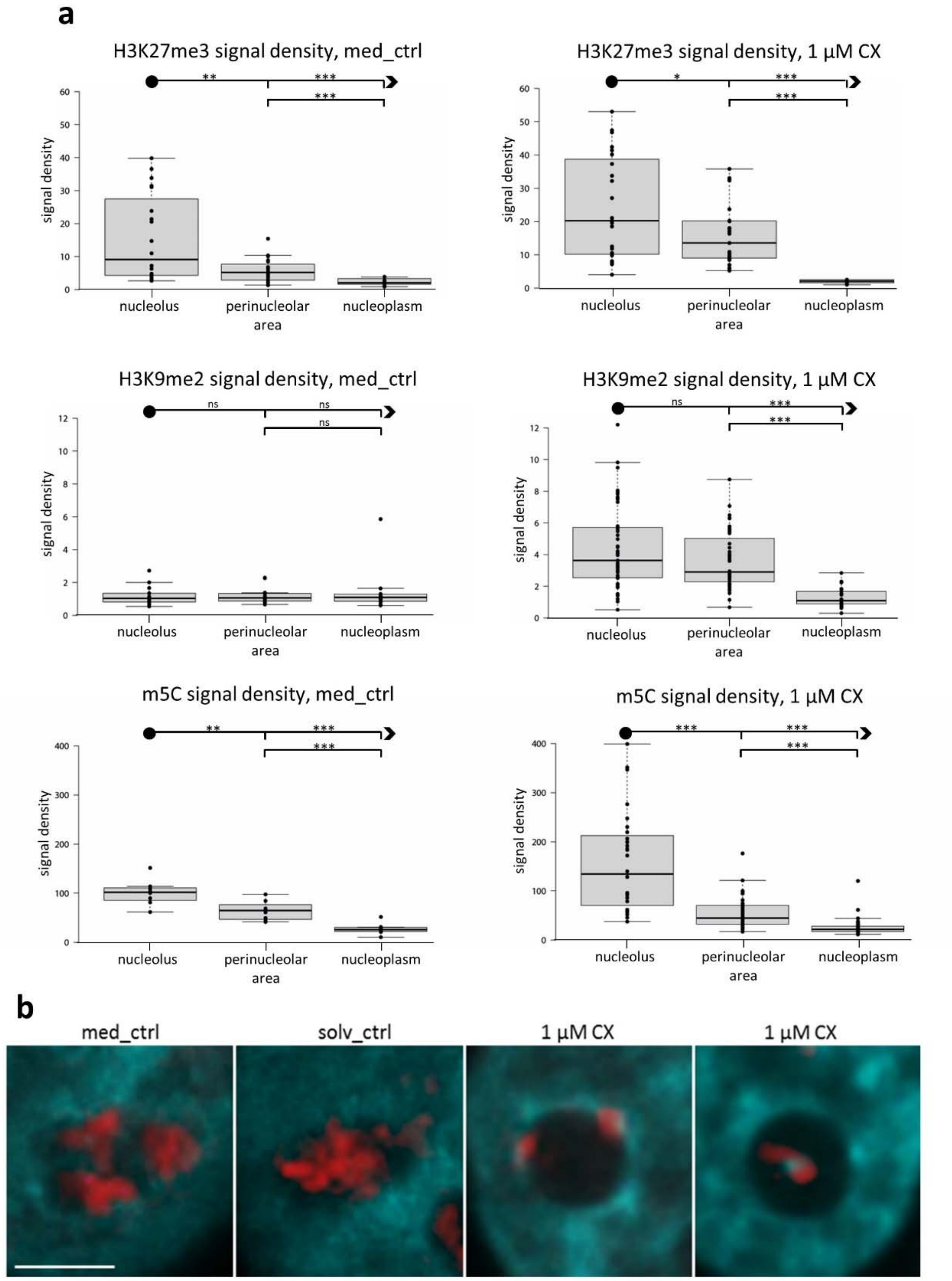

Figure S3. Quantification of DNA methylation and heterochromatin markers (Fig. S2) and representative example of rDNA localization in CX treated versus non-treated cells. (**a**) Measurement of signal densities over nucleoli, perinucleolar area and nucleoplasm in HeLa cells. CX-5461 treatment leads to higher signal densities of DNA methylation (m5C) and heterochromatin markers over nucleoli and for histone modification markers also over perinucleolar chromatin (mean, background-corrected grey values; 25 nucleoli out of 1 experiment (H2K9me2, H3K27me3) and 20 nucleoli out of 2 experiments (m5C) were evaluated); (**b**) representative nucleoli displaying rDNA (red; DAPI blue) in Hep3B cells (med_ctrl and solv_ctrl) show dispersed signal pattern typical for cancer cells; after treatment with CX-5461 cells show reduced signal within nucleoli while more signal can be seen at the perinucleolar chromatin indicative of transcriptionally silent rDNA. Association of rDNA with a nucleolar tunnel can be observed in the rightmost example; single confocal sections. Box plot charts show first and third quartile, sample median and whiskers (1.5 x interquartile range); Student´s *t*-test was applied. Bar= 1 μm; ns= not significant, * p<0.05, ** p<0.01, *** p<0.001.

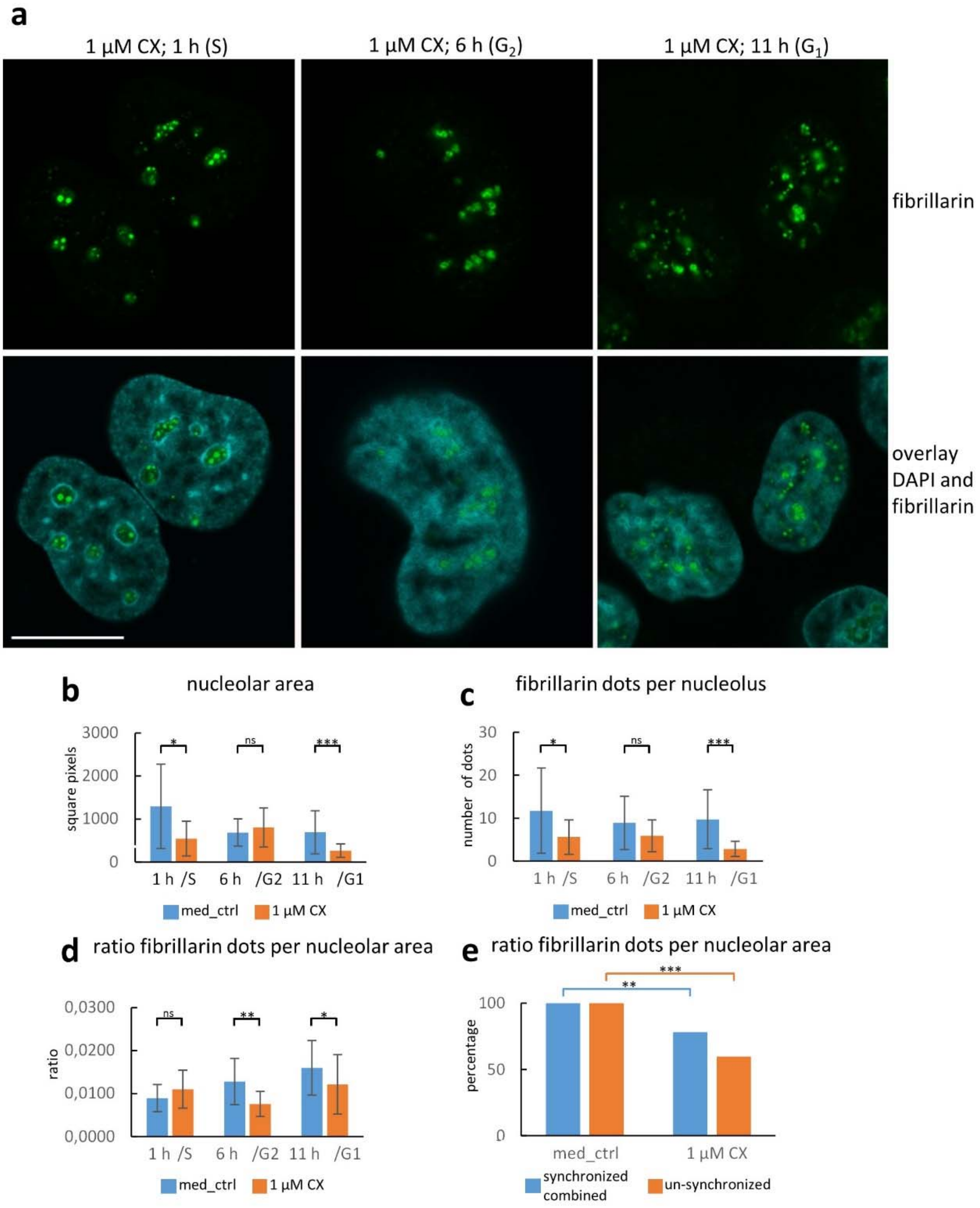

Figure S4: Cell cycle dependency of nucleolar morphology upon CX-5461 administration. Representative images of fixed HeLa cells synchronized by double-thymidine block and incubated with CX-5461 after indicated time intervals when cells progressed into respective phases of the cell cycle (in brackets). (**a**) upper row: fibrillarin (green), lower row: overlay DAPI (blue) and fibrillarin. DAPI and fibrillarin staining show subtle differences dependent on the stage at which CX-5461 was applied (representative cells; single confocal sections); (**b**, **c**) graphs show that the change upon CX-5461 exposure in either nucleolar size or number of fibrillarin dots were strongest in G_1-phase followed by S-phase; (**d**) relating nucleolar size and fibrillarin dot numbers differences were strongest in G_2 -phase followed by G_1-phase; (**e**) combined data of synchronized cells result in significant change of ratios (dot numbers divided by nucleolar area) after CX-5461 exposure, which is in agreement with unsynchronized, exponentially growing cells (i.e. the same dataset as used for Fig. 3d). (b-e) 20 nucleoli per category out of 1 experiment were evaluated. Graph bars display means, error bars represent standard deviations; Student´s *t*-test was applied. Bar= 10 µm; ns= not significant, * $p<0.05$, ** $p<0.01$, *** $p<0.001$.

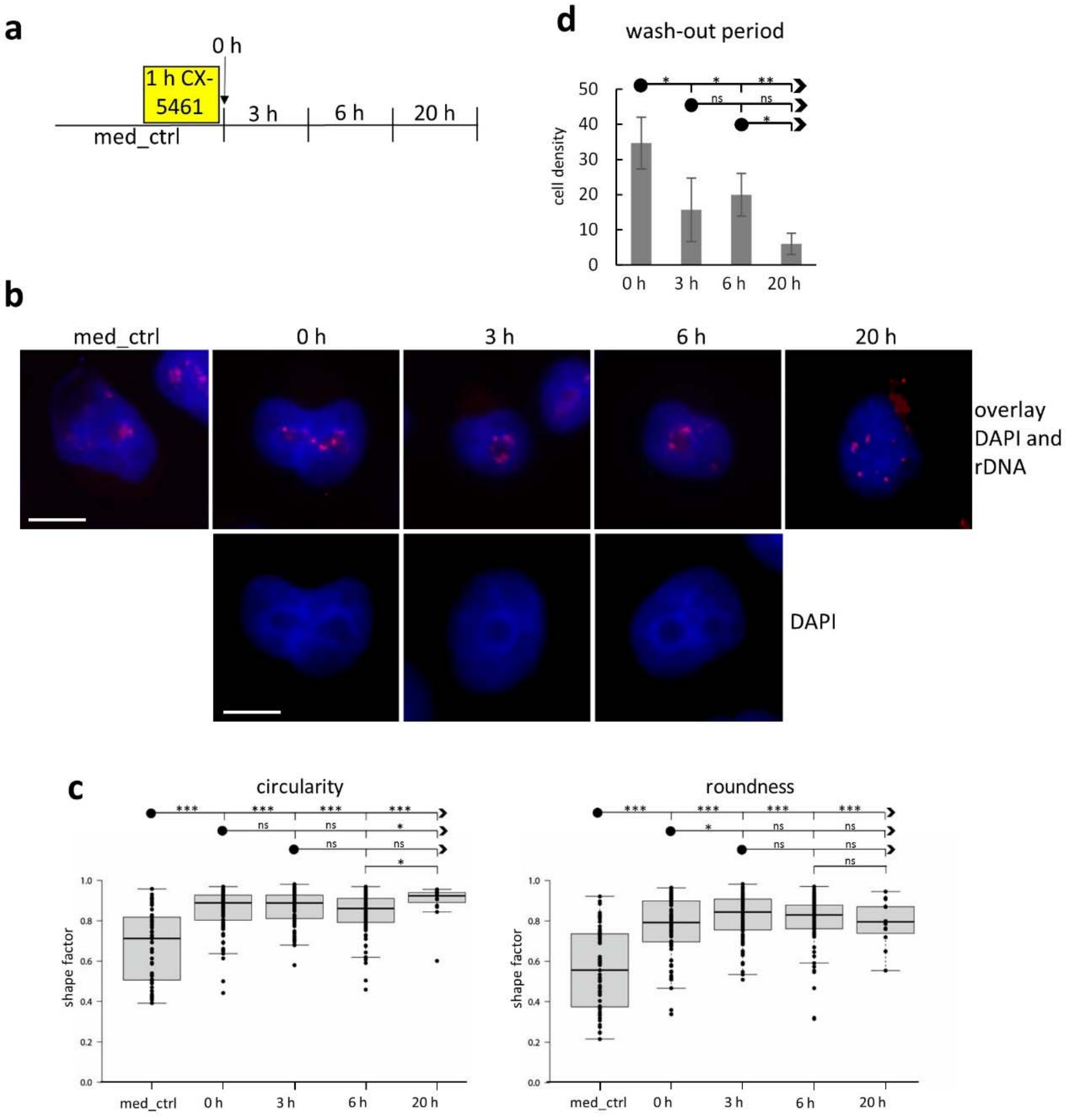

Figure S5: Irreversibility of CX-5461 treatment (“wash-out”) in HeLa cells. (**a**) experimental setup; vertical lines represent time points of fixation; (**b**) upper row: rDNA detection by FISH (red; DAPI in blue) in different stages of wash-out reveals no significant changes in rDNA distribution, lower row: likewise, the DAPI-positive ring persists during stages of wash-out (representative cells; widefield imaging); (**c**) graphs show that morphology factors remain stable during the incubation periods after removal of the inhibitor; (**d**) cell numbers (nuclei per image frame) significantly decrease with time. Bar graph displays means, error bars represent standard deviations; box plot charts show first and third quartile, sample median and whiskers (1.5 x interquartile range); (c,d) 1 experiment; Student´s *t*-test was applied. Bars= 10 µm; ns= not significant, * $p<0.05$, ** $p<0.01$, *** $p<0.001$

Legends for Supplementary Video files

Supplementary Movie 1. Time-lapse imaging of untreated and CX-5461 treated HeLa cells expressing histone H2B-YFP. Left: control (med_ctrl); right: 1 µM CX-5461 treatment. Note decreasing nucleolar size (e.g. middle nucleolus in CX-5461-treated cell) and increase in perinucleolar condensed chromatin. Single confocal sections imaged for 60 minutes (representative cells).

Supplementary Movie 2. Time-lapse imaging of a nucleolus of a HeLa cells expressing histone H2B-YFP during exposure to CX-5461 showing nucleolar tunnel. Upper left: boxed area indicates enlargement seen in the movie (upper right); lower row: selected stills showing nucleolar tunnel (red arrowheads) and its disappearance towards the end of the sequence. Single confocal sections imaged for 60 minutes (representative nucleolus).

Supplementary Movie 3. Time-lapse imaging of untreated and CX-5461 treated HeLa cells expressing histone H2B and RNA polymerase I (pol I). The two cells stably express histone H2B-mCherry (red) and transiently pol I-EGFP (green); for better assessment of nucleolar shape changes, the pol I-channel was omitted in the left column. Upper row: nuclear size and pol I foci numbers are stable in controls (med_ctrl); lower row: note dramatic reduction in number of pol I foci after treatment with 1 µM CX-5461. Single confocal sections imaged for 60 minutes (representative cells).

Supplementary Movie 4. Time-lapse imaging of untreated and CX-5461 treated HeLa cells expressing histone H2B and fibrillarin (fib). The two cells stably express histone H2B-YFP (green) and transiently fib-mCherry (red); for better assessment of nucleolar shape changes, the fib-channel was omitted in the left column. Upper row: nuclear size is stable in controls (med_ctrl); lower row: note drastic reduction in nucleolar size and appearance and disappearance of nucleolar tunnels in the two large nucleoli after treatment with 1 µM CX-5461. Single confocal sections imaged for 60 minutes (representative cells).
